# Understanding Biases in Liquid–Liquid Phase Separation: Investigating Amino Acid Enrichments in Phase-Separating Proteins toward Peptide Design

**DOI:** 10.1021/acs.biomac.4c00224

**Published:** 2025-09-22

**Authors:** Joana Calvário, Diogo Antunes, Rita Cipriano, Daniela Kalafatovic, Goran Mauša, Ana S. Pina

**Affiliations:** † Instituto de Tecnologia Química e Biológica António Xavier, 98819 Universidade Nova de Lisboa, Av. da República, 2780-157 Oeiras, Portugal; ‡ 112563 University of Rijeka Faculty of Engineering, 51000 Rijeka, Croatia; § Center for Artificial Intelligence and Cybersecurity, University of Rijeka, 51000 Rijeka, Croatia

## Abstract

Liquid–liquid phase separation (LLPS) facilitates the formation of membraneless organelles, enhancing biochemical processes. The stickers-and-spacers model explains LLPS but is mainly validated in prion-like RNA-binding proteins. To broaden our understanding, we investigated peptide motifs associated with LLPS across diverse protein contexts using a computational approach on the droplet-promoting regions (DPRs) of 178 phase-separating proteins. The study identified 129 enriched peptide motifs (3–6 residues), characterized by Gly-rich sequences interspersed with aromatic, charged, and polar residues, as well as homopeptide repeats (e.g., GGDR, SRGG, QQQQ). Analysis of motif presence and frequency revealed a widespread distribution across DPRs and significant repetitive patterns. Motif trios with a higher likelihood of co-occurrence were utilized in a data-driven approach to design peptides with LLPS propensity. The designed peptides exhibited liquid-like behavior with different dynamics upon experimental validation. This work provides insights into sequence determinants of phase separation and offers the potential for designing synthetic condensates with tailored properties.

## Graphical abstract

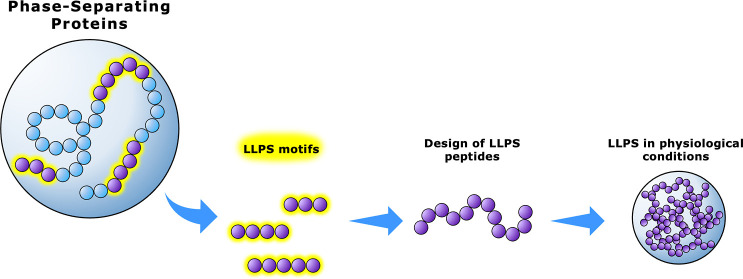

## 1 Introduction

Compartmentalization is a fundamental feature of living systems, allowing for specialized environments through physical membranes or liquid–liquid phase separation (LLPS) and leading to the formation of membraneless organelles, such as nucleoli and stress granules. Such partitioning in cells enhances essential biochemical processes.^1–5^ LLPS-derived organelles often comprise intrinsically disordered proteins/regions (IDPs/IDRs), known for their low-complexity amino acid regions (LCRs) and lack of a defined 3D structure, and nucleic acids.^1–3,6,7^ The sequence composition and distribution of LCRs in IDPs considerably influences phase transitions, prompting recent research into how primary sequences affect LLPS.^3,4,8^

The stickers-and-spacers model, inspired by associative polymers, provides a framework for understanding LLPS in IDPs. “Stickers”, often aromatic amino acids (e.g., Tyr, Phe), interact via noncovalent bonds (hydrophobic, electrostatic, π–π, cation−π, and hydrogen bonds), while “spacers” are typically polar residues (e.g., Gly, Ser, Gln) that provide flexibility and a dynamic nature to the protein.^1–3,5^ Consistent distribution of sticker residues and charged amino acid clusters promotes LLPS, while its absence may lead to amorphous precipitates, highlighting the critical role of multivalency, patterning, and charge distribution in driving protein phase separation.^2,5,9^ The stickers-and-spacers model has proven valuable in understanding LLPS, yet its validation has primarily focused on prion-like RNA-binding proteins (RBPs), particularly the FET family (FUS, EWSR1, and TAF15).^2,4,5,10^ This narrow focus on RNA-binding proteins has limited our understanding of how protein context and function influence LLPS across diverse families, leaving uncertainty about whether such principles can be generalized to other cellular functions. The use of peptide-based model systems in synthetic environments has also been used to study LLPS in a simplified manner, although continuing to focus on the stickers-and-spacers conceptual model.^11–18^

Specific motifs associated with LLPS have been identified through a combination of serendipitous observations, targeted studies of known proteins, and bioinformatic analyses of existing protein databases, including GAR (glycine- and arginine-rich), YGG motifs, and proline-rich regions.^19–21^

Recent LLPS studies employ specialized computational tools. Predictors^22,23^ use machine learning to identify phase-separating proteins (PhSePs); publicly available trained models^24–26^ analyze physicochemical properties, while databases^27–29^ catalog verified LLPS proteins. These resources aid in identifying and characterizing LLPS-prone proteins. Despite significant progress in artificial intelligence and algorithmic approaches for studying LLPS, the context-dependent relationship between sequence and LLPS remains underexplored. Current algorithms often exhibit biases toward specific protein families or motifs, which constrains their predictive capabilities. Furthermore, existing approaches have limitations in their ability to rationally design novel LLPS-prone sequences, as these have been primarily focused on the stickers-and-spacers model.

In this work, we aim to contribute to the understanding of LLPS rules across diverse protein classes, considering both functionality and context, and further design synthetic minimalistic models based on peptide systems. We developed a computational approach for peptide motif discovery using a database of 178 phase-separating proteins (PhSePs), categorized by function. Our approach was complemented by two widely recognized web tools in the field of disordered proteins and LLPS: the FuzDrop method, introduced by Fuxreiter and Vendruscolo,^25,26^ and the classification of intrinsically disordered ensemble regions (CIDER) server, developed by the Pappu laboratory.^24,30,31^ The FuzDrop method is an invaluable resource for elucidating the principles of phase transitions and the likelihood of liquid-like droplet formation through its predictive analysis of local sequence properties such as composition, hydrophobicity, and structural disorder. We utilized FuzDrop to predict the probability of proteins undergoing LLPS and to pinpoint droplet-promoting regions (DPRs) within their sequences. The CIDER server is an essential tool for analyzing intrinsic disorder-related characteristics and has been used to study the distribution of such parameters across all IDRs in the human proteome, providing insights into their potential phase separation behavior.^32^ We used CIDER to examine several parameters in DPRs, including charge distribution, hydropathy, and fraction of disorder-promoting residues, while also comparing these metrics with those of the NODPR regions of PhSePs.

Our approach encompassed the analysis of amino acid composition and their properties in PhSePs and DPRs, as well as the identification and evaluation of enriched patterns of peptide motifs (ranging from 3 to 6 residues in length) through comparison with the same peptide motifs in a negative control database containing proteins that do not phase separate.

We evaluated the presence (number of distinct DPRs containing at least one instance of a peptide motif), frequency (total incidences of a peptide motif across all DPRs, including repeats within sequences), and co-occurrence (number of DPRs where three peptide motifs coexist). While the presence and frequency provide insights about the importance of peptide motif distribution within a sequence, the co-occurrence of the motifs provides the strategy to design synthetic peptides composed of 10 to 14 residues by optimizing the sequence space of the designed minimalistic peptides. This design enabled the incorporation of diverse motif combinations and amino acid distributions, which were subsequently validated experimentally.

## 2 Materials and Methods

### 2.1 Deciphering Amino Acid Motif Patterns in Phase-Separating Proteins

For the present study, we employed two databases, comprising a total of 178 distinct phase-separating proteins (PhSePs) sourced from LLPSDB^27,28^ and PhaSePro.^29^ The selection of these databases was driven by their robust experimental background and thorough validation of the phase separation behaviors. Furthermore, we ensured that any UniProt ID duplicates were removed to avoid redundancies. The PhSePs were classified based on their functionality, including RNA-binding, DNA-binding, chromatin-binding, regulation, hydrolase, and structure proteins, with respective counts of 79, 42, 21, 27, 17, and 10 proteins.

We used the FuzDrop method^25,26^ to identify droplet-promoting regions (DPRs) and regions that do not promote droplet formation (NODPRs). The resulting 712 DPRs in our database were, on average, 72.1 amino acids in length, while the 702 NODPRs had an average of 90.2 residues.

To ensure statistical reliability of our subsequent analysis, we created a negative control data set. This data set was curated from the Universal Protein Resource (UniProt) and consisted initially of 3000 human proteins. Each protein in this data set has a length ranging from 400 to 800 residues, comparable to our primary data set of PhSePs, which have an average length of around 600 residues. We processed these 3000 proteins through the FuzDrop method. Subsequently, we randomly selected 208 proteins that demonstrated a droplet-promoting probability (pDP) below 20%, referred to as non-PhSePs. On average, the 208 proteins from the negative database exhibited 0 to 4 DPRs, with a mean value of 0.92 DPRs per protein.

For the following computational analysis, we used the Python 3.11 programming language, Spyder 5.4.3 integrated development environment, and Anaconda Navigator 2.5.2. graphical user interface. To quantify the composition of amino acid residues and their character, we created the “STATITIAN.py” script, which calculates the frequencies of all 20 amino acids within the full protein sequences and separately within DPRs and NODPRs. This analysis provided both count and percentage distributions of the amino acids along with a detailed account of the side chain properties for each residue. The same method was additionally applied to each protein family, as well as non-PhSePs.

For further analysis of our database, we employed the CIDER (classification of intrinsically disordered ensemble regions) algorithm, specifically using localCIDER Version 0.1.18. This analysis focused on five key parameters: the fraction of charged residues (FCR), net charge per residue (NCPR), kappa (κ), mean hydropathy, and the fraction of disorder-promoting residues. The script utilized in this step, “CIDER FOR PROTEINS”, is available in the GitHub repository.

To study amino acid patterning in PhSePs, we conducted a thorough analysis of all protein sequences, focusing on the DPR regions. We developed the “SELECTOR.py” script to analyze DPR sequences for all possible motifs of a specified length. By providing an input of the target peptide motif length (in our study, we used a length of 3 to 6 residues for a minimalistic approach), our program identifies all contiguous subsequences of the specified length within the DPR database. The script simultaneously computes a motif’s presence (number of distinct DPRs with at least one instance of a motif) and frequency (total occurrences across all DPRs, including repeats within sequences). This program was used in our research for motif discovery in the DPRs of PhSePs, the proteins by family, as well as non-PhSePs. Fold values were calculated to quantify both the enrichment and the frequency of a given motif within the DPR sequences of PhSePs, relative to a negative database comprising non-PhSePs. Specifically, the fold represents the ratio between the motif’s presence/frequency in the DPRs of PhSePs and its presence/frequency in non-PhSePs. The resulting fold values were then normalized on a scale of 0 to 1, thus returning a presence fold (PF) and a frequency fold (FF). Recognizing that both the presence and frequency of motifs are crucial for understanding the mechanism of LLPS, we assigned them equal importance by calculating a combined fold score (CF), where CF = 0.5 PF + 0.5 FF. To identify the most prevalent and frequently occurring motifs, we focused on motifs with a CF of 0.2 or higher. As a result, 129 motifs met this condition. The presence and frequency of the 129 discovered motifs were additionally computed in the NODPRs sequences to validate their enrichment in DPRs.

We developed the “FREQUENCY.py” script, which enabled us to further assess presence and frequency values per individual DPR sequence, both in PhSePs and in protein families.

All of the described scripts can be found in our GitHub repository.

### 2.2 Minimalistic Peptide Design Based on Motif Co-occurrence

To design minimalistic peptides with LLPS propensity based on the co-occurrence of motifs, we developed the “COMBINER.py” script, which generates all possible arrangements of selected peptide motifs. Our analysis specifically focused on combinations of three motifs from the 129 discovered ones, each 3 to 6 residues long, with the aim of creating short peptides under 20 residues that distill key LLPS-promoting elements from full-length sequences while retaining their phase separation potential.

“COMBINER.py” calculates the extent to which three motifs coexist in DPR sequences by computing the co-occurrence of motif pairs (A with B, A with C, and so on) and representing these as vectors. The sum of these vectors (vector score or VS) represents both the number of DPRs in which the three motifs coexist and their symmetry of coexistence. Symmetry in this context refers to the equal occurrence of motif pairs, indicating that motif A co-occurs with motif B as frequently as it does with motif C. This process is repeated for all motif pair combinations, and an average is calculated to produce the final score (FS). For a more detailed mathematical explanation of the “COMBINER.py” script, please refer to the guide in our GitHub repository. This script originated a set of all possible combinations of motif trios, resulting in small peptides with an average of 12 residues. Additionally, we computed a matrix using the script “MATRITIAN.py” that shows the coexistence of the motif trios and their respective FS, which can be found in our GitHub repository.

For the analysis of the designed small peptide sequences, we once again used localCIDER Version 0.1.18, focusing on the parameters fraction of charged residues (FCR), net charge per residue (NCPR), kappa (κ), mean hydropathy, and the fraction of disorder-promoting residues. The program utilized in this step, “CIDER FOR PEPTIDES”, is available in our GitHub repository.

### 2.3 Experimental Validation of Designed Peptides

#### 2.3.1 Materials

The peptides were purchased from GeneCust (France) with 98.0% purity.

PBS tablets were obtained from a ThermoFisher. Pluronics was purchased from Panreac AppliChem. The microscopy material was obtained from Avantor and Zeiss. The 3′,6′-dihydroxy-6-isothiocyanatospiro­[2-benzofuran-3,9′-xanthene]-1-one (FITC) compound was purchased from Sigma-Aldrich.

#### 2.3.2 Trifluoroacetic Acid (TFA) Removal

The peptides were dissolved in a 10 mM HCl solution, incubated for 30 min, and subsequently lyophilized using a LabConco Freeze-Dryer. This process was repeated three times in total to ensure the efficient removal of trifluoroacetic acid (TFA). The final TFA content was verified to be below 1%.

#### 2.3.3 Evaluation of LLPS Propensity and Partitioning Experiments

Lyophilized peptide powders were reconstituted in 1 mL of distilled water and vortexed until complete dissolution was achieved, resulting in transparent solutions. This process was repeated to obtain three different peptide concentrations: 1 mg/mL, 5 mg/mL, and 10 mg/mL. Droplet formation was induced by mixing 60 μL of each peptide solution with 240 μL of PBS.^18^ The sample was incubated for 1 h at 37 °C (±1 °C) and left at room temperature (23 °C ± 2 °C) for 3 h. To investigate the encapsulation of guest molecules within the condensates, we performed partitioning experiments with guest molecule fluorescein isothiocyanate (FITC). Droplet formation was once again induced by mixing 60 μL of each peptide solution with 240 μL of PBS. The sample was incubated for 5 min at 37 °C (±1 °C), followed by the addition of 1 mM FITC. The sample was incubated for 1 h at 37 °C (±1 °C) and left at room temperature (23 °C ± 2 °C) for 3 h. An aliquot of 2 μL was taken from the partitioning sample and diluted with 198 μL of PBS. The remaining sample was then centrifuged at room temperature for 30 min. Following centrifugation, 2 μL of the supernatant was collected and diluted with 198 μL of PBS. The fluorescence measurement of both samples was conducted in the INFINITE M NANO + TECAN microplate reader. The encapsulation efficiency (% EE) was calculated using the equation % EE = (CT – *C*_sup_)/CT, where CT represents the total fluorophore concentration and *C*_sup_ represents the concentration in the diluted supernatant phase.

#### 2.3.4 Turbidity Measurements

Turbidity measurements were conducted using an INFINITE M NANO + TECAN microplate reader. Absorbance at 600 nm was recorded every 5 min for a total duration of 60 min. All measurements were carried out at a temperature of 37 °C (±1 °C). The relative turbidity values reported represent triplicate measurements and were calculated using the formula τ relative % = 100 – *T* % = 100 – [100% × 10^–A600 nm^], where A600 is the absorbance at 600 nm. A well containing an equivalent volume of the buffer solution served as the blank.

#### 2.3.5 Condensate Imaging

For bright-field images, a Leica DM6000B upright microscope equipped with an Andor iXon 885 EMCCD camera was used. The MetaMorph V5.8 software was employed to control the microscope, and the images were acquired using a 63×1.4 NA oil immersion phase-contrast objective (Leica). Image analysis was conducted using ImageJ/FIJI 1.54f.^33^

Confocal images were acquired by using a Zeiss LSM 880 point scanning confocal microscope equipped with photomultiplier tube (PMT) detectors and a gallium arsenide phosphide (GaAsP) detector. To visualize individual condensates, 10 μL of the peptide solution was added to Pluronic-functionalized glass slides. Condensates were imaged using a 63x Plan-Apochromat 1.4 NA DIC oil immersion objective (Zeiss) with laser lines at 405 and 488 nm and appropriate spectral separation for FITC. The Zeiss Zen 2.3 (black edition) software was used to control the microscope and adjust spectral detection for the excitation/emission of the fluorophores used (following the manufacturer’s recommendations). Imaging was performed with 1–2% laser intensity for all lasers and a gain between 500 and 650. Image analysis was conducted using ImageJ/FIJI 1.54f.^33^

#### 2.3.6 Fluorescence Recovery after Photobleaching (FRAP)

Sample preparation was performed as described in Section 2.3.3. For fluorescence recovery dynamics assessment, a prebleached image of the condensates was acquired using a 488 nm laser line excitation and emission collected with a GaAsP detector at 2% power. Subsequently, the target condensates were bleached using the 488 nm laser line at maximum power for 0.45 to 0.93 s. The subsequent recovery of the bleached area was recorded using the same acquisition parameters as the prebleached image, with a total recovery time of 45 s. The final fluorescence recovery after photobleaching (FRAP) recovery curves represent the average of three recovery curves collected from *n* = 3 separate droplets. Corrections for photobleaching, normalization, and averaging were performed using ImageJ/FIJI.^33,34^ The halftime of recovery was calculated as described by Aumiller et al.^35^

## 3 Results and Discussion

### 3.1 Deciphering Amino Acid Motif Patterns in Phase-Separating Proteins

Our first objective was to investigate whether there is a bias of amino acids and peptide motifs in terms of composition, patterning (specific arrangement of residues or motifs), and frequency (repetition of peptide motifs along a sequence) in phase-separating proteins (PhSePs), regardless of their specific function or controlled environments. To this end, we analyzed a cohort of 178 distinct PhSePs, collated from the LLPSDB^27,28^ and PhaSePro^29^ databases. These proteins were classified according to their functions into six families: RNA-binding, DNA-binding, chromatin-binding, regulation (which includes activators, repressors, and other processing-related proteins), hydrolases, and structure (which provides support and integrity to biological structures) proteins. The distribution of proteins across these categories was 79, 42, 21, 27, 17, and 10, respectively. To ensure the validity of our results, we additionally created a negative control database, comprised of 208 proteins with a less than 20% probability of undergoing spontaneous LLPS (referred to as non-PhSePs), as validated by the FuzDrop method. This comparative analysis is key to understanding the sequence-specific factors that drive LLPS.

#### 3.1.1 Amino Acid Enrichment in PhSePs

The amino acid enrichment analysis was performed on PhSePs, encompassing full sequences, droplet-promoting regions (DPRs), and non-droplet-promoting regions (NODPRs). This analysis was also performed on PhSePs grouped by families (RNA-binding, DNA-binding, chromatin-binding, regulation, hydrolases, and structure) as well as on non-PhSePs. The detailed findings can be found in Figures S1–S6, which show the results for all individual sequences, while Figure 2 highlights the averaged obtained data.

**Figure 1:**
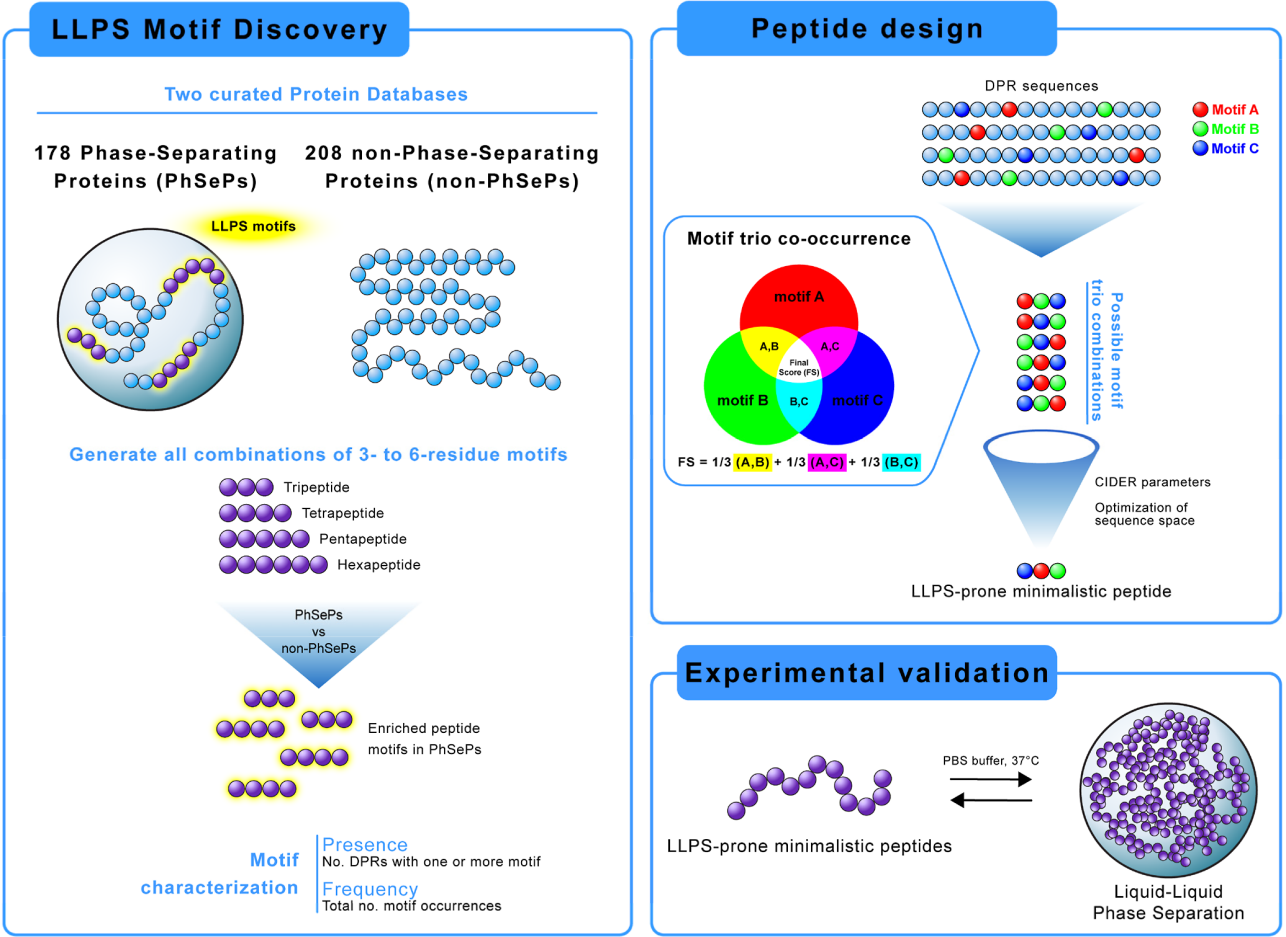
Graphical abstract showcasing our computational approach for LLPS motif discovery and peptide design. The workflow includes (1) analysis of phase-separating proteins for identification and characterization of significant peptide motifs (2–6 residues), (2) data-driven design of minimalistic LLPS-prone peptides by motif co-occurrence studies, and (3) experimental validation through induced LLPS using synthetic peptides.

**Figure 2:**
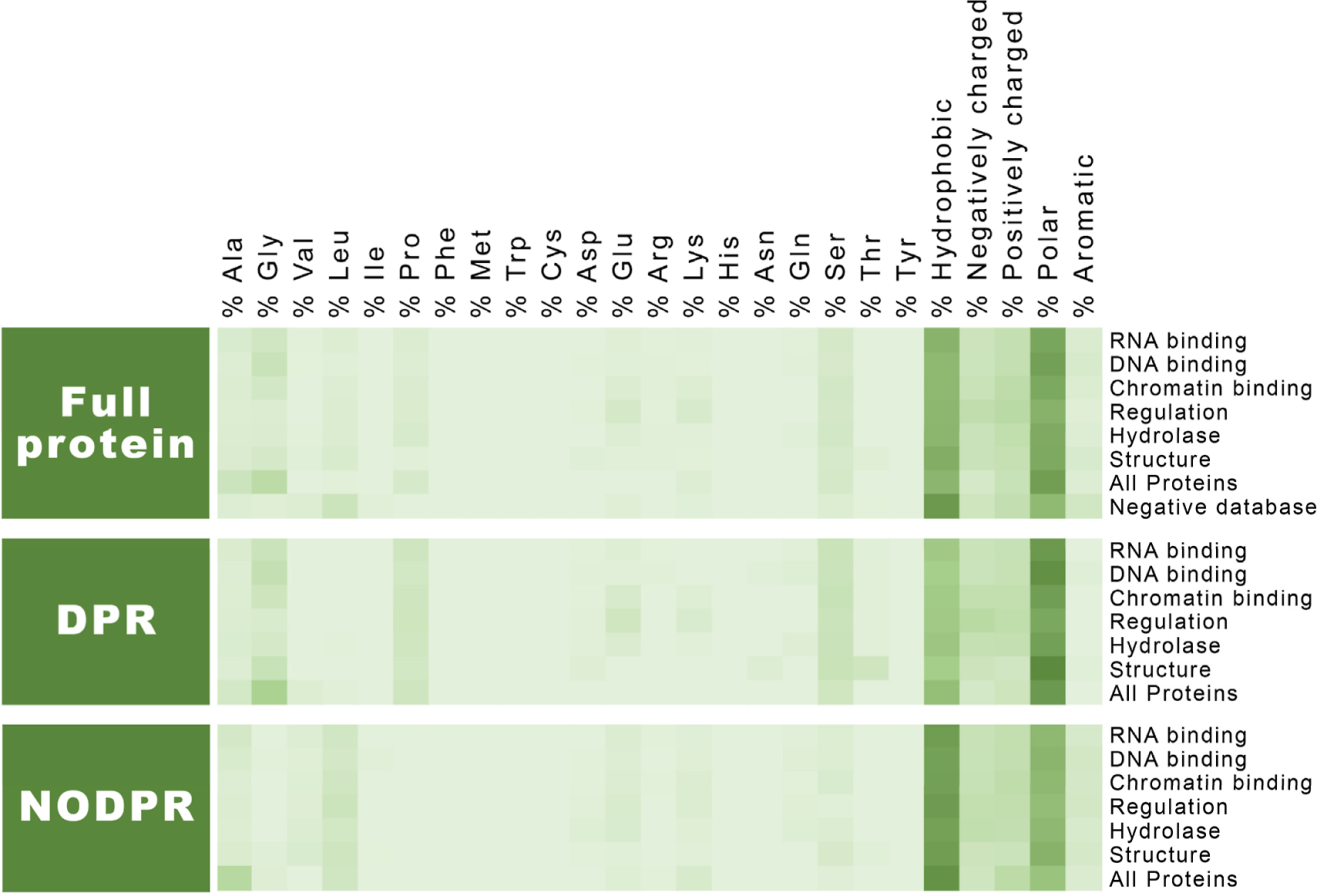
Analysis of amino acids and residue character enrichment in full proteins (including the PhSePs database, PhSePs by family, and the negative database of non-PhSePs); DPRs (PhSePs database, PhSePs by family); and NODPRs (PhSePs database, PhSePs by family). The color gradient transitions from lighter hues (corresponding to 0%) to darker shades (indicating the maximum value of enrichment in this analysis, corresponding to 50%).

A significant disparity is observed when comparing the distribution of amino acids in the full protein and NODPR regions against the distributions found within the DPR regions. Notably, the DPR regions show a distinct over-representation of Gly, Ser, Pro, and Ala. While Gly is not known to directly engage in interactions that lead to LLPS, it is crucial for the dynamics of protein regions in LLPS by maintaining flexible peptide bonds, making it common in disordered regions associated with LLPS.^4,27^

We also observed a heightened proportion of polar amino acids and a reduced proportion of hydrophobic and aromatic residues within the DPR regions. Conversely, NODPRs displayed the opposite trend, characterized by a higher concentration of hydrophobic residues and a lower proportion of polar amino acids. These findings align with prior research indicating that polar residues promote disorder, while small fractions of aromatic and charged residues offer the necessary multivalency for interactions that allow for LLPS.^3,6^

The unique arrangement of aromatic amino acids in specific patterns is vital for the biocondensate formation. They are often interspersed with charged amino acids in mostly polar sequences. While essential for LLPS, excessive aromatic residues can also lead to aggregation.^5,6^ Their π electron-rich rings facilitate π–π stacking, enhancing the stability of biocondensates, while charged amino acids promote phase separation through cation–π interactions.^2–4,9^

When analyzing the amino acid enrichment in the protein data set segmented by family, we note an overall similar pattern (Figures 2 and S3–S6), namely, a prevalence of polar amino acids and the reduced proportion of hydrophobic and aromatic residues in DPR regions.

RNA-binding proteins have a higher prevalence of polar amino acids and are likely to interact with highly charged RNA molecules. Conversely, chromatin-binding proteins are rich in Lys, a positively charged residue that allows for ionic interactions with negatively charged DNA and histones, crucial for chromatin–protein complex formation and regulation of chromatin compaction.^36^

Structure proteins are enriched in Gly, Ser, Pro, Ala, and Val. Gly provides flexibility and stability, properties common to proteins such as collagen, elastin, and keratin.^37–41^ Val contributes to integrity and hydrophobic interactions within the protein and is found in high proportions in elastin, titin, and fibroin,^38,42,43^ while Pro is enriched in collagen and elastin^37,38,40,41^ and Ser in keratin and fibroin.^39,43^ Moreover, Val and Ala are components of repeating motifs found in elastin and fibroin.^40–43^

The enrichment of Ala, Asp, and Thr in hydrolases is logical, since Asp and Thr are crucial catalytic residues, while Ala aids in maintaining enzyme flexibility and accessibility, essential for conformational changes and substrate interactions in catalysis.^44^ The prevalence of these residues in enzymes undergoing LLPS suggests a potential link between amino acids involved in LLPS and catalysis, which is plausible to hypothesize given that LLPS is known to enhance catalytic activity in natural systems.^45,46^

The analysis of the negative database reveals completely distinct trends, including a higher average of hydrophobic and aromatic residues and a lower frequency of polar amino acids. Table 1 underscores that the key residues enriched in PhSePs, such as Gly and Pro, are absent in non-PhSePs, with the exception of Ser. Additionally, non-PhSePs show an over-representation of Leu residues, a pattern not observed in PhSePs.

**Table 1:** Top Enriched Amino Acids Influencing LLPS^a^

protein	enriched amino acids				
literature	Gly	Ser	Thr	Tyr	Gln
full database	Gly (11.5%)	Ser (11.7%)	Pro (10.6%)	Ala (7.6%)	Glu (6.4%)
RNA-binding	Gly (13.2%)	Ser (11.4%)	Pro (9.7%)	Ala (6.6%)	Gln (6.4%)
DNA-binding	Gly (11.0%)	Ser (12.2%)	Pro (10.4%)	Ala (6.9%)	Glu (8.2%)
chromatin-binding	Gly (7.8%)	Ser (11.6%)	Pro (10.8%)	Lys (7.7%)	Glu (10.2%)
regulation	Gly (8.7%)	Ser (11.8%)	Pro (10.2%)	Ala (7.4%)	Glu (7.4%)
hydrolase	Gly (12.1%)	Ser (12.0%)	Pro (9.6%)	Ala (6.7%)	Thr (10.4%)
structure	Gly (19.7%)	Ser (10.1%)	Pro (10.7%)	Ala (9.3%)	Val (6.9%)
negative database	Leu (11.1%)	Ser (6.8%)	Val (6.9%)	Ala (6.6%)	Glu (6.3%)

a Represented is literature-based commonly reported amino acids,^1–4,30^ our database analysis of the DPR regions of 178 PhSePs, protein family-based analysis of DPR, and negative database analysis on non-PhSePs.

Having conducted a comprehensive analysis of the amino acid characteristics in DPRs and NODPRs, we sought to explore whether we could differentiate between the properties of the primary sequences of DPRs and those of NODPRs. To accomplish this, we utilized the CIDER server,^24^ a powerful tool designed to analyze parameters associated with the primary sequence of IDPs, thus giving insights about the behavior of unstructured ensembles.^24,30–32^ For the analysis of DPRs and NODPRs, we focused on five key metrics used in the comprehensive work of Ginell and Holehouse,^32^ who calculated these across all IDRs in the human proteome. The parameters are the fraction of charged residues or FCR (proportion of charged amino acids), the net charge per residue or NCPR (overall charge of the sequence, considering positive and negative charges), kappa or κ (quantifies the extent of charge mixing along the sequence), mean hydropathy (reflects the hydrophobicity of a sequence), and the fraction of disorder-promoting residues (proportion of amino acids predicted to be disorder-prompting, here encompassing Ala, Arg, Gly, Gln, Ser, Pro, Glu, and Lys).^24,31,32^ The resulting graph plots obtained from analyzing the DPR and NODPR sequences using these parameters are presented in Figure S7.

Both charge-based parameters (FCR and NCPR) show similar trends between DPRs and NODPRs, falling within the range observed for human IDRs.^32^ This aligns with our previous amino acid analysis, which revealed no significant difference in the overall charge content among the full PhSePs, DPRs, and NOPPRs. Although DPRs may contain repeating and enriched motifs with charged amino acids, we propose that NODPRs exhibit a similar charge content without the same repetitive patterns, resulting in comparable overall charge percentages. Consequently, these charge-based parameters alone are insufficient for distinguishing between DPRs and NODPRs.

Charge clustering and segregation are recognized as important factors in LLPS, with clustered charges promoting such phenomena.^24,27,47^ Therefore, it is not surprising that the κ parameter, which measures the charge distribution, differs slightly between DPRs and NODPRs. For DPRs, the κ value falls within the range of human IDRs,^32^ while for NODPRs, it does not. The higher value for DPRs (where κ closer to 0 indicates more distributed charge and closer to 1 indicates more clustered charge) indicates a more clustered presence of charged residues. This parameter provides a means to distinguish DPRs from NODPRs and further associates DPRs with known characteristics of sequences prone to LLPS.

Hydropathy is a measure of the relative hydrophobicity or hydrophilicity of the amino acids in a protein sequence.^48^ This metric can be used to differentiate DPRs from NODPRs, where hydropathy values for DPRs fall within the range observed for human IDRs (corresponding to lower hydrophobicity), while the value for NODPRs exceeds the upper limit of this range. This is corroborated by the literature, which highlights that IDPs are known to have lower hydrophobic content compared to structured proteins.^24,32,49^

DPRs also exhibit a higher proportion of residues involved in LLPS, with a value within the range observed for human IDRs.^32^ In contrast, NODPRs have a lower proportion of such residues with a value outside the IDP range. This clear distinction in LLPS-prone residue content provides a robust metric for differentiating between DPRs and NODPRs.

Our analysis aligns with the known literature regarding amino acid enrichment patterns in PhSePs. Although minor variations exist among different protein families, such differences are consistent with their specific functionalities (e.g., catalytic residues in hydrolases), indicating that the propensity of a protein to undergo LLPS may also depend on its specific family context. Regarding sequence differences between DPRs and NODPRs, several metrics usually used for IDPs can be used to distinguish between these. The main differentiating factors include those related to charge patterning, hydrophobic content, and the proportion of residues associated with disorder.

#### 3.1.2 Motif Discovery in PhSePs

Amino acid patterning, which refers to the arrangement or organization of specific amino acids in motifs, is also fundamental in LLPS.^1–3,5,8,9,30,50^ After the analysis of the DPR and NODPR regions of all PhSePs, it became evident that NODPR regions exhibit a prominently randomized distribution of residues. In contrast, DPR regions tend to display significantly more consistent and enriched patterns.

To investigate which patterns are enriched in our DPR database, we comprehensively examined all peptide motif combinations ranging from three to six amino acids in length by computing their presence (number of distinct DPRs with at least one instance of a motif) and frequency (total occurrences across all DPRs, including repeats within sequences), represented in Figure 3A. To ensure the validity of the obtained values, we repeated the same process using our negative database of non-PhSePs. The addition of a negative database for data correlation is key to understanding the sequence-specific factors that drive LLPS, as we aim to study motifs enriched in proteins that undergo phase separation, rather than in proteins in general.

**Figure 3:**
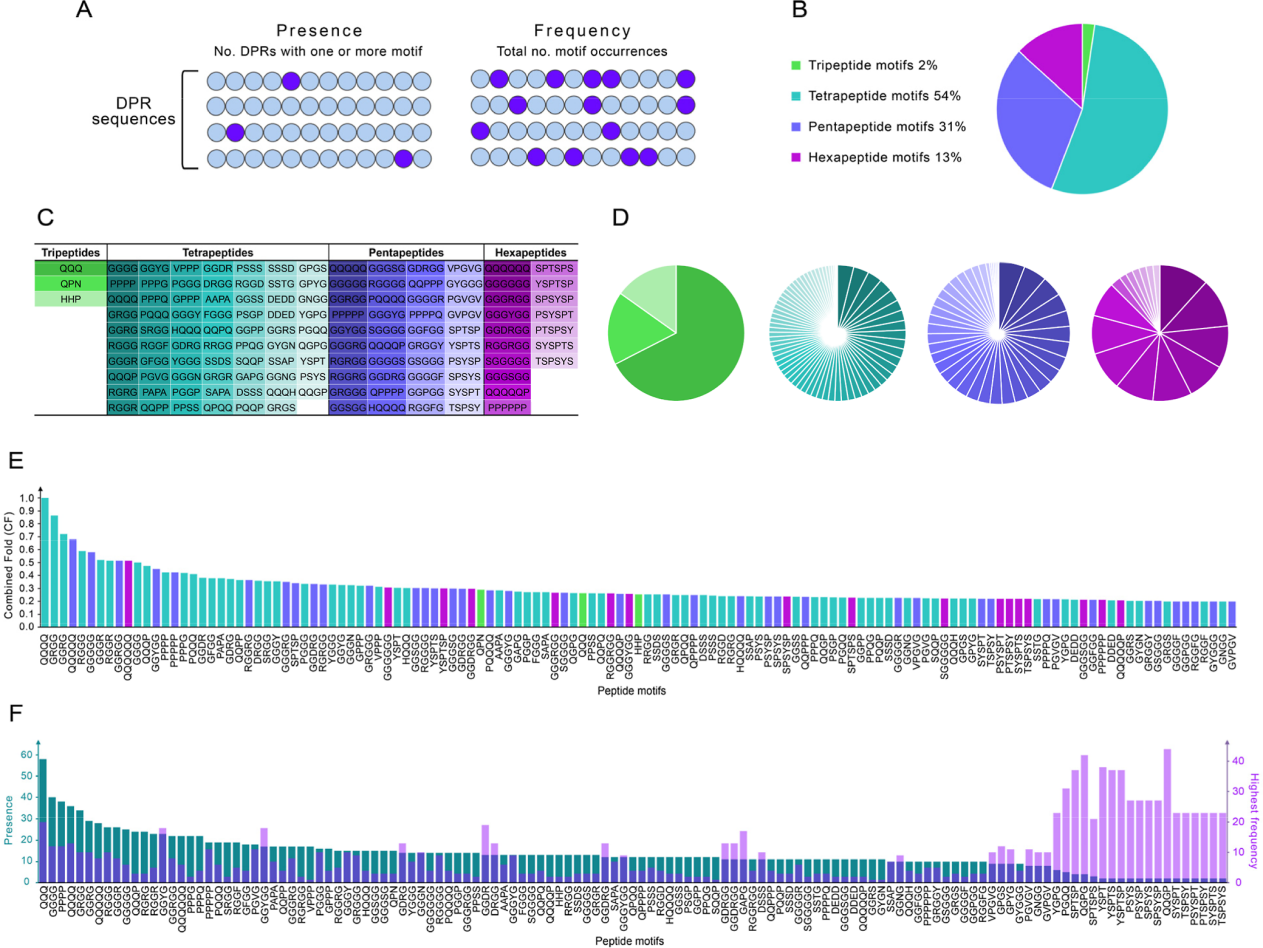
Motif discovery analysis of PhSePs. (A) Schematic representation of the presence and frequency parameters; (B) distribution of the 129 discovered peptide motifs by length, ranging from tripeptides to hexapeptides; (C) listing of the 129 identified peptide motifs; (D) proportion of enriched peptide motifs based on their presence values depending on peptide lengths; (E) distribution of peptides based on their CF values; and (F) presence values of the motifs organized in descending order, along with their highest frequency value observed in any DPR.

Motif relevance was assessed using a combined fold score (CF) that equally weighted the normalized presence fold (PF = presence in DPRs of PhSePs/presence in non-PhSePs) and normalized frequency fold (FF = frequency in DPRs of PhSePs/frequency in non-PhSePs) of each motif. Both PF and FF, as well as CF, have normalized values between 0 and 1. Motifs with a CF ≥ 0.2 were considered, resulting in 129 motifs for further analysis, as illustrated in Figure 3C.

From the 129 motifs identified, 36% were found to be embedded within others (e.g., QQQQ in QQQQQ, GRGG in GGRGG), with the majority of these being embedded in only 1–2 other motifs. We chose not to eliminate any of these instances because we consider that even a single amino acid addition may significantly impact the LLPS behavior that we are studying.

To validate that the enrichment of these 129 motifs in DPRs differs from that in NODPRs, we also analyzed the motif presence and frequency in the NODPR sequences. The maximum value for motif presence in NODPRs was 18 with a frequency of 19, while in DPRs, the presence was 58 with a frequency of 199. The presence and frequency values for all motifs are provided in Table S1.

#### 3.1.3 Characterizing Motif Composition and Diversity in PhSePs

Among the resulting 129 peptide motifs, the length distribution includes 2% tripeptides, 54% tetrapeptides, 31% pentapeptides, and 13% hexapeptides (Figure 3B). This distribution suggests that tetrapeptides might serve as the fundamental unit for LLPS and could represent an optimal balance between length and interaction potential, contributing to the stability of phase-separated states.^15,51^ In Figures 3C and 3D, the distribution of the 129 peptide motifs is illustrated regarding their presence in the DPR sequences and organized by peptide length. Based on average PF and FF values, tetrapeptides are the most present, followed by tri- and pentapeptides. While hexapeptides are less common overall, they appear with higher frequencies in DPR sequences, followed by tetra- and pentapeptides. This indicates that shorter peptides are generally more enriched in the DPR database, while longer peptides, although less abundant, appear to show more repetitions in fewer sequences. Figure 3E further shows the distribution of discovered motifs based on CF values.

Among the discovered enriched motifs, individual amino acid trends are observed. Gly is notably overrepresented, appearing in 59% of motifs, followed by Pro (47%), Ser (29%), Arg (23%), Gln (20%), and Tyr (19%). When comparing the amino acid enrichment in the peptide motifs with the overall enrichment in DPR sequences in Figure 1, we observe that Gly, Pro, and Ser are the top three residues in both analyses, while Arg, Gln, and Tyr showed no enrichment in DPR sequences. In fact, Tyr ranks among the bottom five amino acids present in DPRs. This suggests that specific arrangements of amino acids within short peptide motifs, composed of polar (Gly, Ser, and Gln) and hydrophobic (Pro) residues and interspersed with charged (Arg) and aromatic (Tyr) residues, may be the key for a minimalistic approach to LLPS of small biomolecules.

Further trends can be found in the set of 129 motifs. First, distinct clusters of homopeptides are present, with notable repetitions of Gln (3 to 6 residues), Pro (4 to 6 residues), and Gly (5 to 6 residues), which are characteristic of low-complexity regions (LCRs) within IDPs. Gln and Gly are prominently represented within LCRs,^6,8^ while Pro is believed to contribute to the nuanced structural dynamics of IDPs.^52,53^ The enrichment of these homopeptides in PhSePs compared to non-PhSePs suggests their key role in enhancing the flexibility and dynamics critical for phase separation, highlighting their greater importance in proteins that undergo LLPS. Interestingly, our analysis revealed not only such clusters but also similar sequences with additional residues interspersed or flanking these clusters, such as QQQP, SGGGG, HQQQ, and GGGR. These additional residues possibly fine-tune the LLPS propensity of proteins by providing specificity, modifying interaction strengths, or influencing the physicochemical properties of the resulting biomolecular condensates.

Charged amino acids are known for facilitating a variety of interactions both within and between proteins.^8,50^ In our analysis, several motifs contain charged residues, primarily positively charged Arg and negatively charged Asp. Interestingly, Arg consistently appears with Gly, typically in a 1:2 to 1:5 ratio (e.g., RGGF, GGRS, GRGGY, and GGGRGG). A subset of Arg containing motifs also includes the oppositely charged Asp (e.g., DRGG, GDRG, and GGDRGG). Motifs containing His are always paired with either Gln or Pro (e.g., HHP, HQQQ). Remarkably, entirely negatively charged motifs (with Asp and Glu) can be found in our set (e.g., DDED, DEDD). Motifs with Asp often co-occur with Gly or Ser (e.g., GGDR, SSDS). Such patterns of charged amino acids with specific residues appear to be important for PhSePs. These recurring combinations likely play key roles in facilitating electrostatic interactions and hydrogen bonding at protein interfaces. The prevalence of Gly in many of these motifs may provide conformational flexibility to optimize the charge residue positioning for intermolecular contacts.

The RG/RGG motif, which is a GAR (glycine- and arginine-rich) motif, is arguably one of the most well-known motif patterns related to LLPS.^12,20,54–56^ This sequence, prevalent in RNA-binding proteins (RBPs), is involved in protein-RNA interactions through Arg–Gly-mediated cation−π, π–π, hydrogen-bonding, and electrostatic interactions.^12,55^ By driving LLPS of several proteins in cells (i.e., FUS, EWS, TAF15, etc.), the RG/RGG motif allows for the formation of membraneless organelles that aid in the regulation of several cellular functions, including ribosome biogenesis and mRNA regulation.^47,48,50^ Interestingly, the RGG motif itself did not appear among the motifs that we discovered. This is due to it being equally present in the non-PhSePs database, leading to a lower fold value compared with motifs that are more enriched in PhSePs. However, we did identify several variations of patterns where the RGG motif was incorporated (a total of 19 motifs), such as DRGG, GRGG, RGGF, GRGGY, GGDRGG, and even a double variation of the motif, RGGRGG. This finding suggests that, while RGG domains can undergo LLPS, the context of specific additional amino acids, consistently identified in our results as Asp, Arg, Gly, Ser, Phe, Tyr, or combinations of up to three of these, may significantly influence condensate formation in synthetic systems.

Aromatic residues, particularly Tyr and Phe, play crucial roles in protein–protein interactions in LLPS, despite their relatively low abundance previously reported. Such residues engage in π–π stacking and cation–π interactions, contributing to multivalent interactions essential for the formation of biomolecular condensates.^4,5,9,57,58^ In our discovered motifs, the prevalence of Gly residues surrounding the aromatic amino acids is striking, with Tyr-containing motifs (e.g., GGGY, GRGGY, and GYGN) and Phe-containing motifs (e.g., FGGG, GGFGG, GGGGF, and RGGFG) being particularly abundant. The number, composition, and patterning of aromatic residues are extremely important in condensate formation and aggregation inhibition, as well as in determining the properties of the droplet, including diffusion rates, biochemical stability, and material phase.^5,58^

YGG motifs can form dense droplets in the context of RNA- and DNA-binding proteins such as hnRNPA2,^20^ FUS, and RBM3.^54^ Such motifs facilitate protein–protein and protein–nucleic acid interactions, with Tyr engaging in π–π stacking and cation–π interactions, while Gly provides flexibility. Similarly to the RGG motif, we did not find the isolated form of the YGG motif but several variations that incorporated additional Gly residues, including YGGG, GGYG, GGYGG, GYGGG, and GGGYGG. Furthermore, our analysis uncovered several other motif variations containing the aromatic Tyr, specifically GPYG, YGPG, and GYGN. Of particular interest, we identified the GRGGY motif that appears to be a hybrid of the RGG and YGG patterns. Again, we report that although the YGG motif unit is known to undergo LLPS, more complex variations with additional residues and patterns might further elucidate the LLPS mechanism and dynamics.

We identified the VPGVG short peptide, as well as adjacent motifs, VPPP, PGVG, PGVGV, and GVPGV, which show partial matches with motifs commonly found in disordered elastins, proteins known for their phase transition capabilities.^40–42^ These proteins typically contain repetitive motifs that allow for their phase separation behavior, such as the canonical pentapeptide VPGVG, as well as variations like VPGG and GVGVP.^38,40–42^ In these motifs, Val residues provide hydrophobicity, Gly offers flexibility, and Pro introduces conformational constraints.^38,41,42^

One notable category of small peptides in our database consists of 7 motifs (YSPT, SPTSP, TSPSY, YSPTS, PTSPSY, SPTSPS, and YSPTSP) and 6 motifs (DRGG, GDRG, GGDR, GDRGG, GGDRG, and GGDRGG). These peptide motifs are embedded within YSPTSPSY and YGGDRGG, which are known periodic repeats present and enriched in PhSePs (RNA polymerase II and TAF15, a member of the FET family of RBPs) that form condensates.^59–61^

Repetitive motifs in PhSePs are essential for LLPS, as their recurring patterns within a protein sequence significantly modulate phase separation. It is thought that a uniform distribution of specific residues induces LLPS, whereas the absence of this particular distribution can lead to the formation of amorphous precipitates.^2,5,9^ Hence, we aimed to examine the presence of motifs (number of DPRs containing a motif) and motif frequency (occurrences across all DPRs, including repeats within sequences), as well as compare the interplay between the two parameters in the discovered motifs. The resulting data in Figure 3F displays the overall presence of the motifs organized in descending order, alongside their highest frequency value observed in a single DPR.

The analysis reveals an equilibrium between the presence and frequency of motifs in the PhSePs. On average, motif presence is 3 to 4 times higher than frequency, suggesting a balanced distribution of these elements across different proteins, with some exceptions visible, as is the case for the motifs GGYG, GGYGG, GDRG, DRGG, GGDRG, GGGYGG, GDRGG, GGDRGG, GAPG, DSSS, SSAP, GGNG, and GVPGV. This suggests that these motifs, when present in a protein, tend to occur with higher repetitions within the same sequence.

Conversely, the right side of the graph (Figure 3F) highlights motifs that, despite their limited presence across proteins, display a high repetitiveness within specific sequences. Notably, partial sequences of the periodic repeat YSPTSPSY demonstrate some of the highest frequency values among the motif list (appearing between 23 and 38 times within a single DPR sequence). Additional highly repetitive motifs, YGPG, PGQQ, QGPG, and QQGP, have frequencies ranging from 23 to 44 repetitions in a DPR. Higher motif repetitions in these proteins might imply a distinct mechanism in LLPS, where multiple copies of specific motifs are more crucial than in typical PhSePs.^62^

Although most of the enriched peptide motifs align with previous results, thus validating our methods, we have also identified several new motifs. These include HHP, QPN, PAPA, AAPA, SAPA, GAPG, GPGS, QQPP, QGPG, PSGP, PPQG, PPSS, SSDS, SSAP, DSSS, DDED, and DEDD. These newly identified motifs could potentially play a significant role in phase separation, thereby expanding our understanding of the sequence determinants of this phenomenon.

#### 3.1.4 Family-Specific Motif Variations

RNA-binding proteins (RBPs) are the most extensively studied proteins undergoing LLPS.^12,20,54–56^ Research has revealed that specific sequence motifs drive LLPS by mediating multivalent interactions, aligning with the stickers-and-spacers model. However, this model has been primarily validated in Prion-like RBPs.^1,2,5^ To determine whether motif enrichment is a general LLPS feature or function-specific, we expanded our analysis to diverse protein families beyond RBPs. We compared motif enrichments across these families to determine if LLPS-associated motifs are function-dependent or represent a broader, function-independent phenomenon.

We performed a similar analysis as done previously, now on the protein family subdatabases, encompassing peptide motif discovery and characterization, while using non-PhSePs as a negative control. Figure 4A depicts motif length distribution, while Figure 4B illustrates motif presence in descending order, alongside their highest frequency within a DPR.

**Figure 4:**
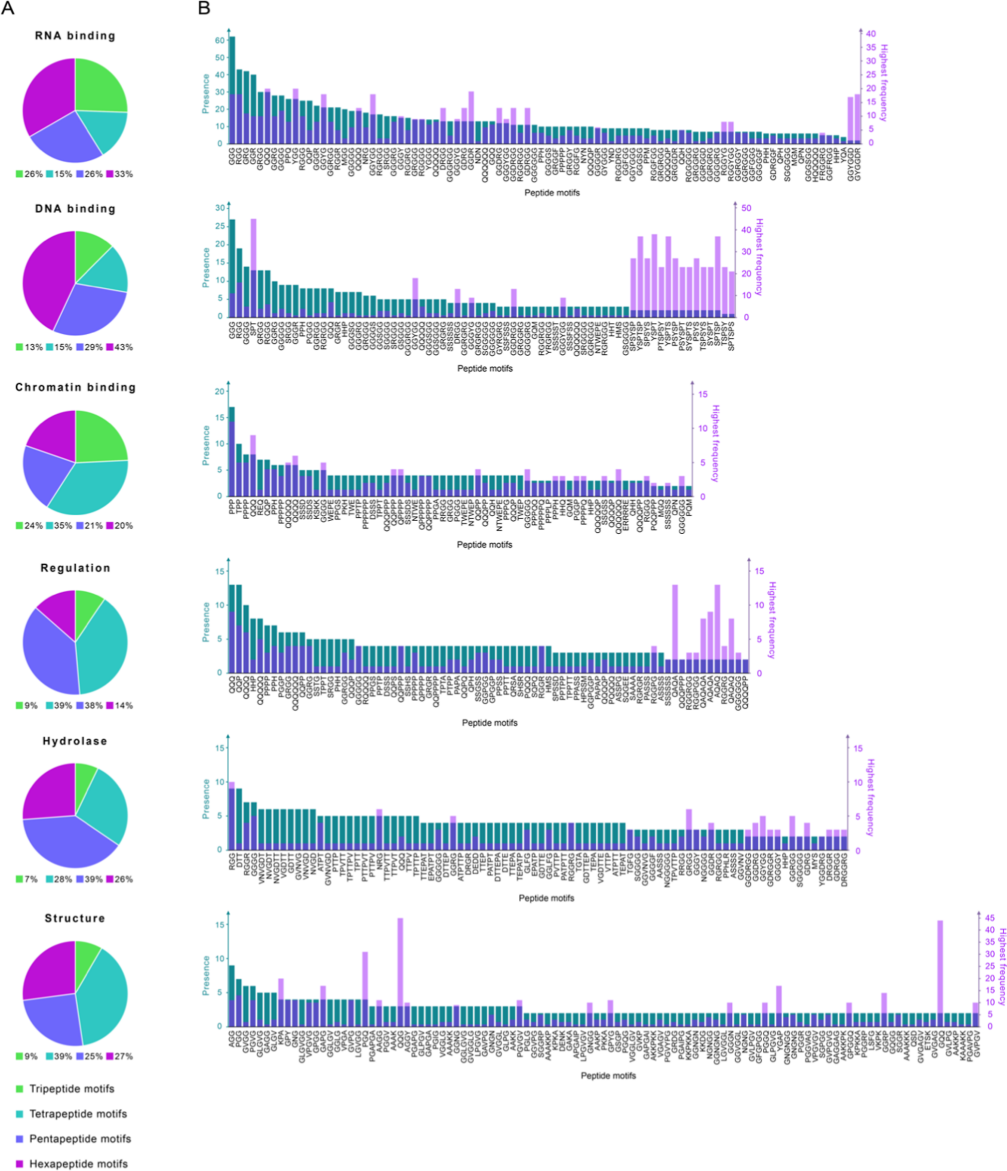
Motif discovery analysis of PhSePs segregated by family. (A) Distribution of the discovered peptide motifs by length, ranging from tripeptides to hexapeptides. (B) Presence values of the motifs organized in descending order, along with their highest frequency value observed in any DPR.

Comparative analysis of motif length distribution across protein families revealed distinct patterns, with tripeptides showing significant enrichment (7–26%) compared to only 2% in full PhSePs, while tetrapeptides, dominant in PhSePs (54%), were underrepresented in families (15–39%). Pentapeptides maintained a relatively high prevalence, while hexapeptides exhibited notable enrichment compared with PhSePs (13–72%).

Regarding distinct families, RNA- and DNA-binding proteins favor longer penta- and hexapeptides, chromatin-binding proteins show a slight preference for shorter tri/tetrapeptides, while regulation proteins prefer midlength tetra- and pentapeptides. Hydrolases are enriched in pentapeptides, followed by tetra- and hexapeptides, and structure proteins favor tetrapeptides, closely followed by penta- and hexapeptides.

Analysis of the amino acid composition in discovered motifs across protein families revealed both conserved trends and function-specific patterns. While the general amino acid enrichment trend persisted, specific amino acids and their ratios vary depending on the functional context. The ubiquity of Gly and Pro suggests their universal role in phase separation, independent of protein function; in contrast, Tyr and Ser, which are enriched in general PhSePs motifs, showed a limited presence in family specific motifs. The nuanced differences in other enriched amino acids likely reflect context-dependent molecular interactions crucial for each family’s specific function.

Three motifs (HHP, GRGG, and GGGGG) are present in five out of six families, excluding the Structure family, while several others (e.g., QQQ, PPH, GGGG, and GGRG) appear in four families. The prevalence of Gly-rich and Pro-containing sequences highlights the role of structural flexibility in LLPS, indicating a common mechanism across cellular functions. Notably, 40–50% of motifs are unique to binding and regulation families, 73% to hydrolases, and 99% to the structure family, suggesting that some motifs contribute to LLPS in general, while others seem to be intrinsically linked to specific functions. The unique motifs of each protein family are listed in Table S2.

In RNA-binding PhSePs, a notable number of motifs are RG/RGG-adjacent patterns,^12,20,54–56^ including RGGGG, FRGGRG, GGRGGD, GRGGDR, and GRGGY, as well as YGG and FGG patterns,^20,54^ such as YGG, RGGYGG, GGYGGD, and GGFGG. We also identified clusters of Gln and Gly, along with versions with additional amino acids like HQQQQQ, GGGGR, and GGGGGF. The discovered motifs show an overall balance between the presence and frequency parameters (Figure 4B), with some outliers (e.g., QQQ, YGG, GGYG, GGYGG) exhibiting higher frequencies than presence. Interestingly, motifs such as GGYGGD and GYGGDR have a low presence but extremely high frequency, appearing 17 and 18 times in a sequence, respectively.

In the DNA-binding category, we observed YSPTSPSY-derived motifs (YSPTSP, SPSYS, PSYSP),^59,60^ as well as RG/RGG patterns (RGRGGG, SRGGG, YRGRGG). Clusters of Gly, Ser, and Gln acid residues were also prominent in our analysis, and notably, we observed many motifs composed solely of Gly and Ser (i.e., GGSGG, GSGGG). These Ser/Gly-rich patterns may be related to the [G/S]­Y­[G/S] domain found in the LCRs of the FUS protein.^20,63,64^ Analysis of Figure 4B reveals that the motif presence and frequency are balanced, similar to the RNA-binding proteins, although with slightly lower frequency values. A notable exception is the motifs related to the periodic repeat YSPTSPSY, which appear to be highly repetitive, with frequencies 11 to 23 times higher than presence values.

Several motifs with positively charged residues were found in chromatin-binding proteins, such as HQQ, PKH, PPPH, KSKK, and ERRRRE. Chromatin has been shown to undergo LLPS, driven by the interactions with positively charged histone tails, a chromatin-binding protein.^65,66^ We also found many motifs enriched in Gln and Pro, likely due to their role in promoting multivalent interactions important for chromatin-associated protein LLPS.^66,67^ However, the specific contribution of Gln/Pro-rich motifs to chromatin binding in LLPS contexts is not well-established in the current literature. The motifs found in chromatin-binding proteins display a close ratio between the presence and frequency parameters.

The motifs enriched in the regulation family, which are Gln-rich sequences (PQQQ, RQQQQ, QQPQ, QAQAQ), are associated with transcriptional activation domains;^68–70^ Gly-rich motifs (GGPGG, GPGGP, RGGPG, RGRGR) are observed in dynamic protein assemblies involved in transcriptional regulation and signal transduction.^19,71^ Pro-rich motifs (PAPAP, PPTPP, PPTT, PPSS) mediate crucial protein–protein interactions,^21,68,70,72^ and Ser-rich sequences (SPSSD, SSTG, SHSR, ASSPG) are phosphorylation sites for activity regulation.^70,73,74^ The repetitive nature of amino acids in motifs (QAQAQA, AQAQA, PAPAP) may suggest a contribution to the structural flexibility required for dynamic regulatory interactions.^70,75^ Interestingly, most motifs in Figure 4B are generally more present than frequent, except for those on the right side of the graph (e.g., QAQA, AQAQ), which consist solely of Gln and Ala and are 4 to 6 times more frequent than they are present.

Enriched motifs in hydrolases contain amino acids typically found in catalytic triads of hydrolases, such as the His in PPHLR or Asp and Glu in DEDD, DTTEP, VGDTTE, and GDTTEP.^44,76^ Aromatic residues in motifs like GGLFG, TGFG, and MYS can be significant for protein–protein interactions, which are important in both enzymatic function and LLPS behavior.^77,78^ We observed a high proportion of motifs enriched in Pro, Val, and Thr that seem to have a repetitive nature (PTTPVT, TPTTPV, TTPVTT, PTTPV, TTPVT, and PVTTP, among others), indicating they may be part of larger elements that contribute to the protein’s architecture and phase separation.^3,30^ By analyzing Figure 4B, we note that motifs in the hydrolase family are 3 to 6 times more present than frequent. However, there are outliers with higher frequencies such as GRGG, GGDRGG, and GGYGG.

In the structure family, we observe a prevalence of Val/Pro-enriched motifs, including GAVPG, GVLPG, GVPG, VPGA, and VPGVG, among others. These sequences align closely with the motifs VPGVG, VPGG, and GVGVP, characteristic of elastin-like polypeptides (ELPs). The abundance of these motifs highlights their essential role in providing elasticity, overall structure, and the ability to undergo phase transitions.^38,40–42^ In structure proteins, motifs are both highly present and frequent, with QQG and GQQ motifs showing exceptionally high frequencies of 45 and 44 occurrences, respectively.

Our analysis revealed diverse LLPS-associated motifs across protein families, indicating unique preferences likely shaped by specific functional requirements, thus underscoring the complex relationship among sequence, function, and phase separation behavior. The varied distribution of motif lengths and presence and frequency values across families suggests distinct mechanisms for LLPS.

### 3.2 Minimalistic Peptide Design Based on Motif Co-occurrence

Our design of minimalistic peptide sequences with LLPS propensity relied on the co-occurrence of enriched motifs, which can create synergistic effects that potentially modulate a biomolecule’s ability to undergo LLPS.

We explored all possible combinations of three motifs from the 129 identified PhSePs (Figure 5). For each trio of motifs, we studied their co-occurrence patterns within DPR sequences by examining how often pairs of motifs appear together (A with B, A with C, and B with C) and the symmetry of their presence (for a more detailed mathematical explanation of our approach, refer to the guide in our GitHub repository). This analysis led to a scoring system (final score or FS) that reflects both the frequency and balance of motif co-occurrence. A perfect score of FS equal to 100 would indicate that all three motifs are present in all DPRs in equal proportions. Scores closer to 50 might suggest either an asymmetrical presence of one motif pair over another or a limited overall presence across DPR sequences. Scores approaching 0 indicate minimal to no co-occurrence of the three motifs. A matrix was computed showcasing the co-occurrence patterns and respective scores for all possible motif trios and can be found in our GitHub repository.

**Figure 5:**
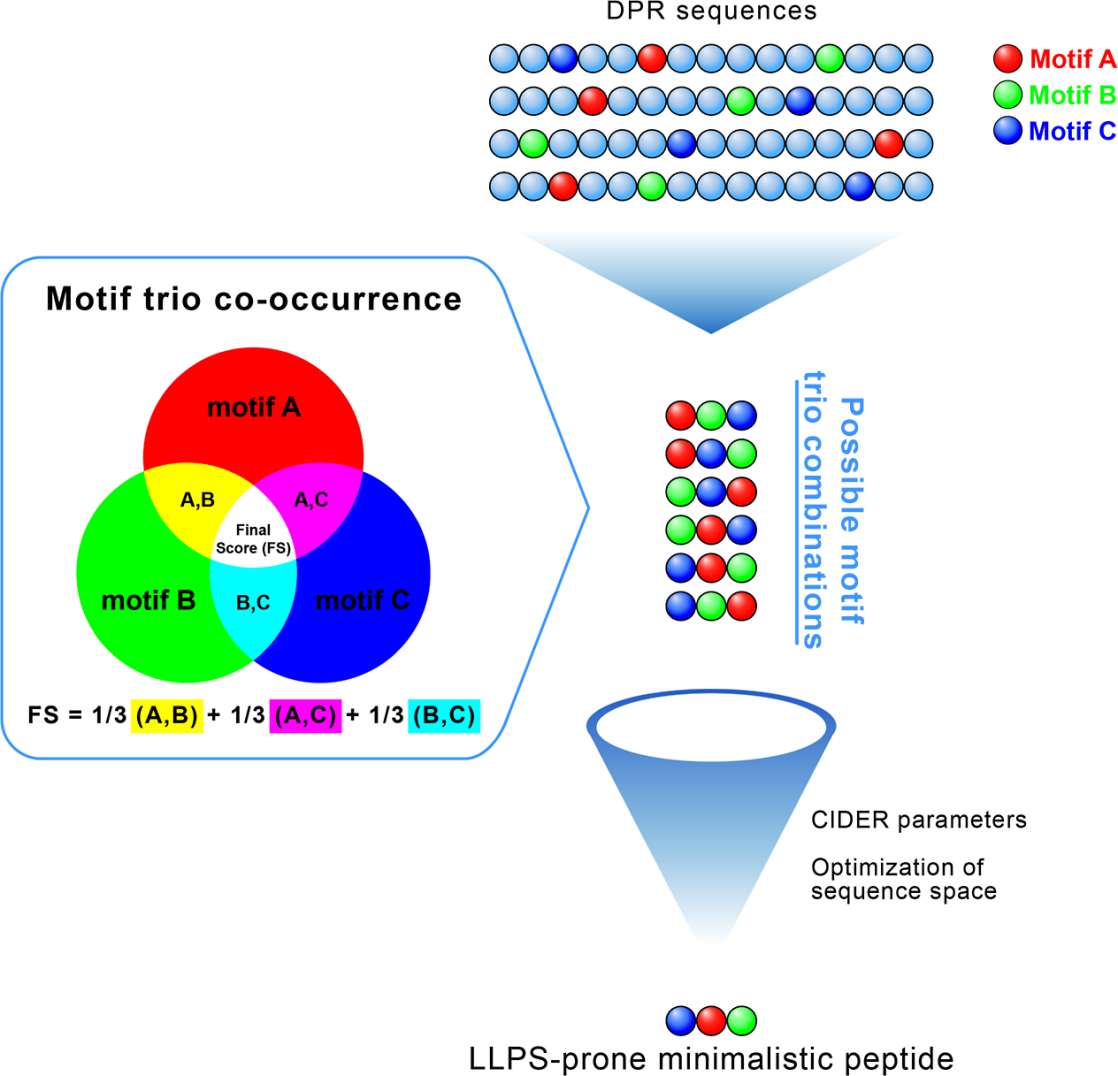
Conceptual approach to peptide design. We analyzed motif trios from the 129 discovered motifs in the context of DPRs, focusing on their presence and symmetry of co-occurrence patterns. All permutations of these motif trios were computed, assigned scores based on their co-occurrence analysis, and ranked by their final scores before further refining them using CIDER parameters and sequence space optimization. This process yielded a list of short peptide sequences with a high potential for LLPS.

We subsequently generated all possible permutations of each motif trio, thus yielding a diverse set of short peptide sequences. These initial designs were refined by identifying and merging overlapping regions between adjacent motifs, eliminating redundancies, and minimizing the sequence length, resulting in peptide sequences with an average length of 12 residues.

Our analysis of the top 10 short peptides ranked by FS reveals a notable over-representation of motifs composed of Gly and Arg residues, followed by motifs rich in Gly interspersed with Phe, Ser, Tyr, Asp, His, and Glu. While these amino acids are known to contribute to LLPS through various mechanisms, our deterministic methodology provides novel insights into their precise combinations and patterns.

While the top-ranking peptides were predominantly composed of Gly and Arg, an examination of the top 100 motif combinations reveals a broader spectrum of amino acid compositions and patterns. These include Pro- and Gln-rich sequences (PPPG, PPPPQ, QQPPP, QQQH, and QQQQ), as well as Ser-rich sequences (GGRS, GGGGS, and GSGGG). This expanded set of motifs underscores the diversity of sequence patterns that may contribute to phase separation properties, suggesting that a wider range of amino acid combinations plays significant roles in LLPS beyond the most prominent Gly–Arg pairings.

To refine our selection of designed peptides from the diverse motif trio permutations, we utilized the CIDER server to compare their characteristics with those of known human IDRs, thereby enhancing our understanding of how these motif arrangements align with typical IDP features.^24,30–32^ We focused on the same key parameters used previously (Section 2.1), drawn from the comprehensive work of Ginell and Holehouse,^32^ calculated in IDRs of the human proteome. Our list of peptides was thus refined and filtered using the range of values for the parameters FCR, NCPR, κ, mean hydropathy, and fraction of disorder-promoting residues and organized according to the FS scores. The distribution of these parameters in the designed peptide sequences is shown in Figure S8.

We generated a peptide library (Table 2) featuring diverse motifs. To ensure a comprehensive representation of our motif discovery results, we intentionally selected peptides that did not share the same motifs, allowing us to explore a broad spectrum of structural and functional possibilities.

**Table 2:** Peptide Sequences Obtained by a Computational Approach

peptide	sequence	final score (FS)
PJ1	FGGGRGGFGGDRGG	74.00
PJ2	GRGGYGGDRGGYGG	61.26
PJ3	GYGGGFGGDRG	60.51
PJ4	GYGNDRGGSGGGG	55.26
PJ5	GPYGDRGGFG	29.36
PJ6	PGVGGYGDRGG	20.90
PJ7	RGGFVPPPRGGD	17.87
PJ8	YSPTSPGGDRGGFG	14.87

We designed 8 peptides (PJ1–PJ8) to explore the impact of amino acid composition on phase separation propensity. PJ1, the peptide with the highest FS score from our co-occurrence analysis, serves as a baseline with Gly, Arg, and Phe. Subsequent peptides introduce the following variations: PJ2 substitutes Tyr for Phe, allowing us to compare the effects of the different aromatic residues. PJ3 incorporates both Tyr and Phe, providing insight into the interplay of multiple aromatic amino acids. PJ4 introduces polar residues Asn and Ser, potentially altering the hydrophilicity and hydrogen bonding capacity of the peptide. PJ5 and PJ6 feature Pro and Val, respectively, allowing us to examine the impact of a cyclic amino acid and a hydrophobic residue on disorder propensity. PJ7 stands out with its Pro triplet (Pro–Pro–Pro) and the strategic placement of charged residues (Arg and Asp) separated by Gly, potentially influencing the peptide’s conformational flexibility and stability. Lastly, PJ8 incorporates a partial sequence of the established LLPS-associated YSPTSPSY motif, serving as a benchmark for the phase separation propensity. Additional notable differences include varying peptide lengths, from 10 to 14 residues, and different patterns of Gly distribution, which may affect the overall peptide flexibility.

### 3.3 Experimental Validation of Designed Peptides

Using the custom-designed peptides, our aim was to evaluate their capability to form condensates under physiological conditions (PBS buffer, 37 °C), focusing solely on the inherent ability of the primary sequences to undergo LLPS. To investigate this, we assess the ability of the PJ peptides to form condensates at three different concentrations (1, 5, and 10 mg/mL). We characterized the resulting samples through optical brightfield microscopy and relative turbidity measurements (Figure 6).^18,79^

**Figure 6:**
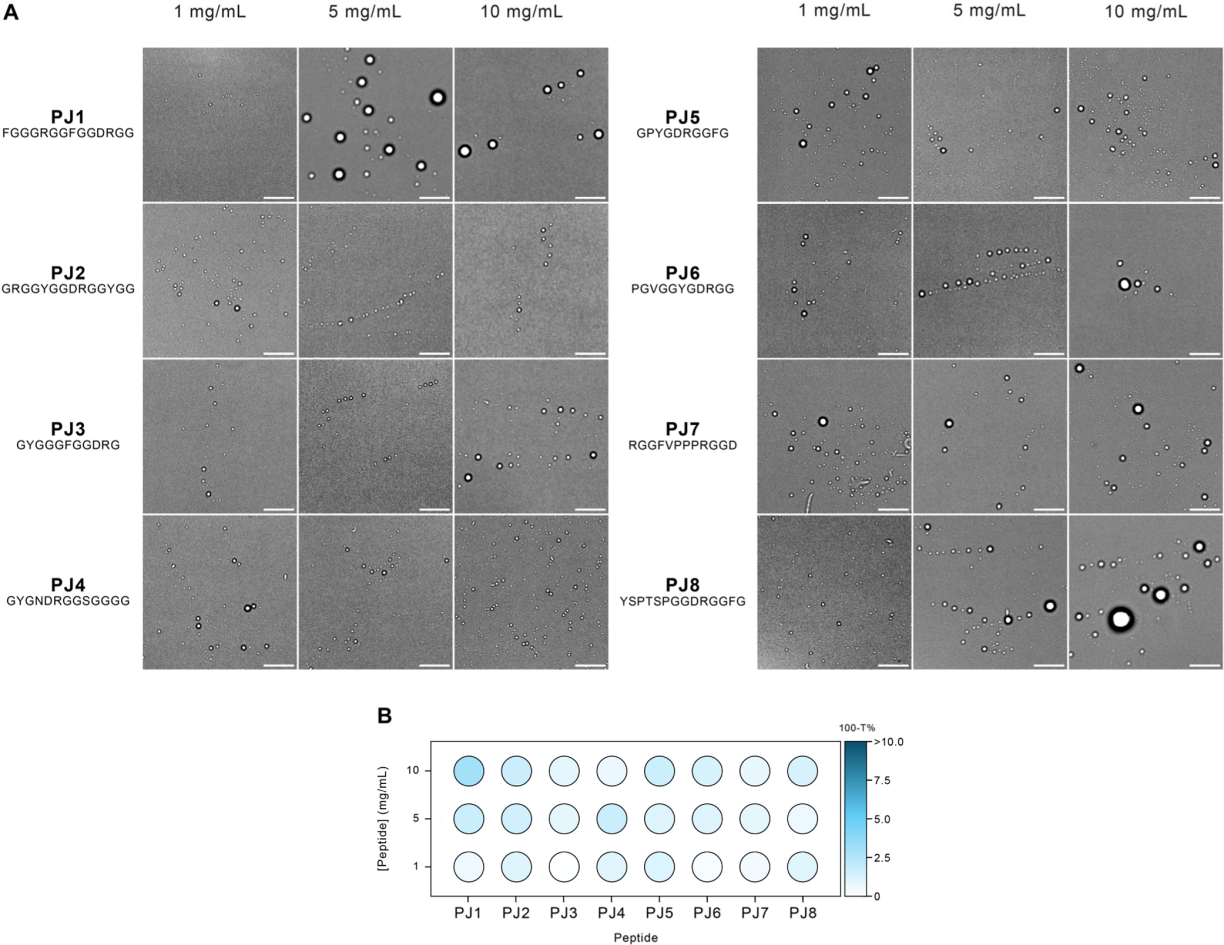
(A) Bright-field microscope images depicting peptide-induced LLPS at various concentrations. Scale bar: 10 μm. (B) Phase diagrams illustrating the effect of different peptide concentrations on the LLPS of PJ peptides, measured by the relative turbidity (100 – *T* %). Different blue shades represent different relative turbidity levels.

Our observations revealed that all samples produced droplets across the full range of peptides and concentrations tested. In our turbidity measurements, we observed a general trend of increased turbidity at higher concentrations across all of the samples. Notably, PJ1 had one of the most effective droplet formations and the highest turbidity.

PJ1, our highest-scoring designed peptide, exhibited enhanced condensate formation at a concentration of 5 mg/mL, with notable abundance and size of the liquid droplets (2–5 μm), in line with previous studies in literature.^12,14,15,18^ This result is particularly significant as it demonstrates that our peptide design method’s top score indeed corresponded to the best LLPS performance.

The PJ8 peptide, containing a partial sequence of the established LLPS-associated YSPTSPSY motif, exhibited an enhanced droplet formation as well. However, PJ8 was not selected primarily for its high score in our design method; its LLPS behavior likely originates from a mechanism distinct from PJ1’s co-occurrence of motif trios. This finding shows an alternative mechanism for LLPS in designed peptides, which presents a promising design strategy for future research. The remaining peptides, despite having different final scores, all undergo LLPS, which is expected since all of our sequences contain LLPS-prone motifs.

Biocondensates are characterized by highly dynamic environments that allow molecular exchange with the surrounding phase, as well as internal mixing.^14,18,30,35^ To investigate the partitioning potential of guest molecules within our droplet systems, we performed an encapsulation efficiency (EE) quantification with a FITC fluorophore. This analysis quantified the proportion of FITC partitioning into the condensates by measuring its relative concentration within the more dense droplet phase compared with the surrounding phase. The peptide-based coacervates exhibited an EE ranging from 15% to 35% across all samples (Table 3), indicating a moderate level of FITC partitioning into the dense phase. The slight variation in EE among peptides might be attributed to distinct interactions between the various peptide sequences and the FITC molecules.

**Table 3:** FRAP and Partitioning Data for PJ Peptide Condensates

peptide	encapsulation efficiency % (EE)	recovery %	halftime of recovery (s)
PJ1	35.6	47.8	2.4
PJ2	20.7	55.2	4.3
PJ3	15.7	58.0	3.3
PJ4	30.3	35.8	2.5
PJ5	25.4	91.9	2.6
PJ6	33.6	76.9	3.6
PJ7	15.1	53.6	1.4
PJ8	28.0	70.4	5.2

Fluorescence recovery after photobleaching (FRAP) was implemented as a powerful technique to study the molecular dynamics and liquid-like character of the peptide condensates by partitioning the FITC guest molecule and performing fluorescence recovery of bleached droplets over time.^18,35,79^ Full droplet FRAP was performed to evaluate the exchange dynamics between the condensate droplets and the surrounding supernatant phase. In this approach, the entire droplet is bleached, requiring molecular exchange with the surrounding dilute phase.^35^ As noted by Aumiller et al., full droplet FRAP can result in lower recovery rates, which can be attributed to the less concentrated dilute phase surrounding the droplet.^35^ To ensure comparability across all PJ peptides, we maintained consistent FRAP parameters (i.e., laser intensity, gain settings, etc.) for all experiments.

The FRAP experiment results for the PJ peptide sequences provide evidence for their liquid-like character, as all peptides exhibited recovery percentages ranging from 36% (PJ4) to 92% (PJ5), as shown in Table 3 and Figure 7. This overall recovery across the peptide set confirms their intrinsic ability for molecular diffusion and local rearrangement within their interior.^26,59,79,80^ The wide range of recovery percentages observed suggests that while all peptides display liquid-like behavior, their specific sequence compositions significantly influence their mobility and diffusion dynamics.

**Figure 7:**
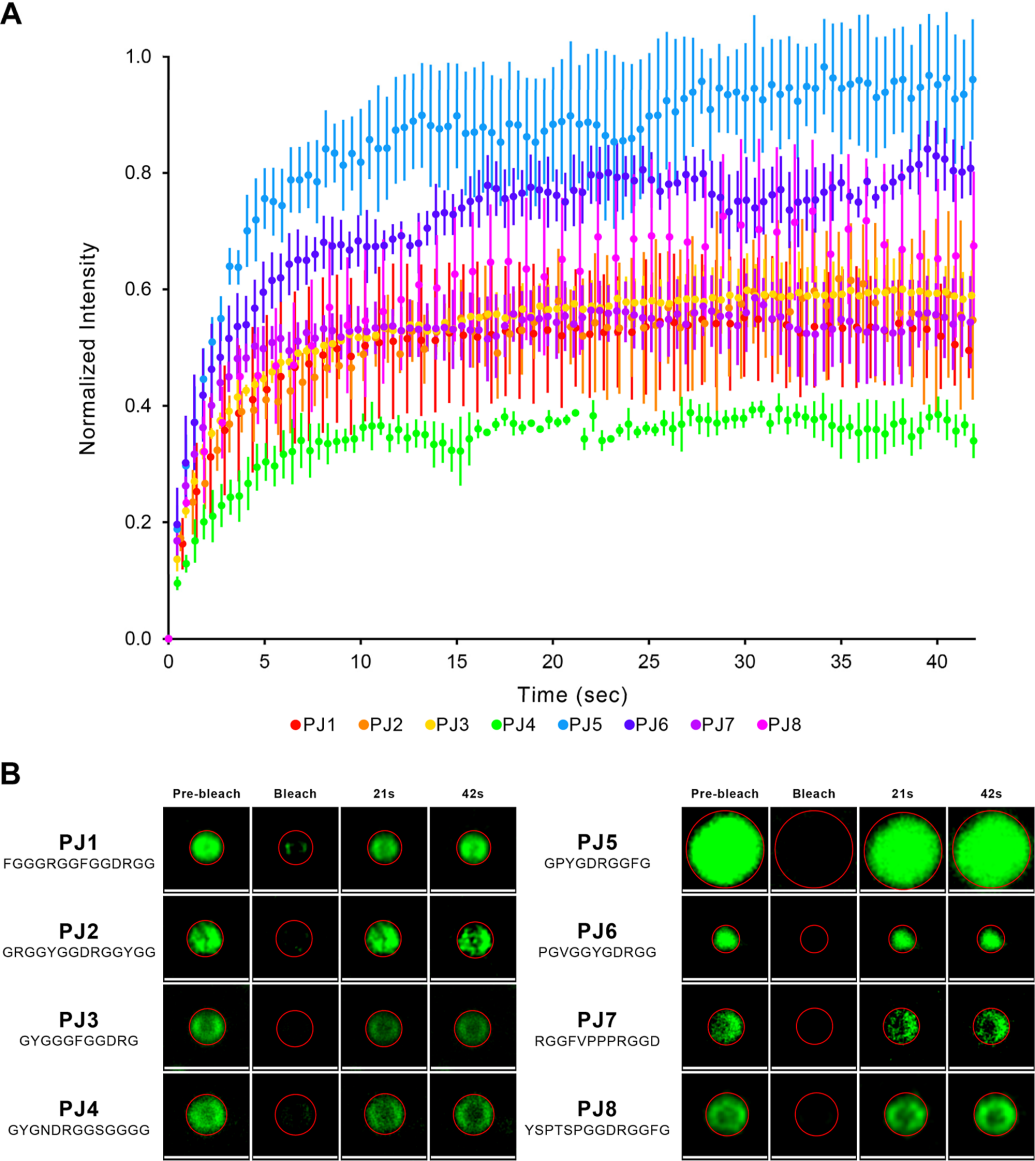
Ability of designed peptides to undergo LLPS and form liquid-like droplets. (A) FRAP analysis of peptide condensates (data are presented as mean values ±SD of 3 condensates). (B) Representative confocal images illustrating the FRAP process in an individual condensate at different time points. Scale bars set to 2 μm.

Among the analyzed peptides, PJ5, the shortest at 10 residues, exhibited the highest recovery percentage at 92%. Its sequence composition and characteristics reflect a balanced combination of features from the entire PJ peptide set, including Gly and Pro relative content, as well as polar and hydrophobic properties—key factors that are known to contribute to LLPS.^3,4,8^

By categorizing the PJ set into peptides with the highest scores in our peptide design method (PJ1–4, scores 55–74) and those with the lowest scores (PJ5–8, scores 15–29), as detailed in Table 2, we can identify notable trends. Peptides PJ1–4 exhibit recovery percentages of 36% to 58%, while PJ5–8 shows higher recovery rates at 54% to 92%.

The lower recoveries of PJ1–4 peptides, which were designed using motifs that frequently co-occur in DPR sequences, suggest that these motifs may engage in inter/intra-molecular interactions, which benefit the formation of a more stable and less flexible network within the droplets.^62,81,82^ Such stability could reduce the fluidity of the droplets, thereby limiting the exchange of molecules between the droplets and their environment.^83^

In contrast, the droplets formed by PJ5–8 peptides exhibit enhanced liquid-like characteristics, as confirmed by their higher fluorescence recoveries. These fluid structures are characterized by rapid organization, facilitating the easy entry, diffusion, and exit of macromolecules,^18,30,80,84^ which can be attributed to reduced constraints from interactions, as low-scoring peptides contain motifs that do not frequently co-occur within DPRs.^30,62,82,83,85^ As a result, fluorescence in laser-bleached regions within these droplets can recover quickly due to this more efficient molecular exchange.^30,62,83,84^

Interestingly, PJ5–8 are the only Pro-containing peptides, which could be another factor that explains the improved fluorescence recoveries. Pro, with its unique propensity to form a cyclic structure, is known to disrupt secondary structures in proteins.^53,86^ However, in small peptides, Pro can induce folding rather than disrupt it by creating a distinctive kink that can promote turns or bends.^12,53,87–91^ This structural change can influence droplet dynamics by promoting peptide folding, which has been shown to enhance the liquid-like character of condensates.^18,92^ This increased fluidity would lead to higher fluorescence recoveries in Pro-containing peptides.

The halftime of recovery, which quantifies the time required for fluorescence intensity to reach 50% of its maximum value,^35^ was calculated and found to range between 1 and 5 s (as seen in Table 3). These values align with previously reported data for similar systems.^35,59,82,84,87,93^

The experimental data validate our computational peptide-design strategy, with all PJ peptides demonstrating LLPS behavior across various concentrations. The FRAP experiments further show the liquid-like nature of our condensates, revealing complex relationships between sequence features and droplet dynamics.

## 4 Conclusions

This study presents a novel approach for identifying and characterizing LLPS short motifs across diverse phase-separating proteins. Our analysis revealed a complex landscape of LLPS-associated sequences, encompassing both known and previously unidentified motifs. The discovery of family specific motif variations highlights the intricate relationship between the protein function and phase separation propensity.

We developed a deterministic computational method for minimalistic peptide design based on the co-occurrence of the discovered motifs in droplet-promoting regions of phase-separating proteins. By selecting a diverse array of peptides generated through this method, we confirmed that all of them undergo LLPS, albeit potentially through different mechanisms. Notably, the different primary sequences resulted in distinct liquid droplet dynamics and behaviors, underscoring the influence of sequence composition on the phase separation characteristics.

Our research contributes to bridging the gap between primary structure and phase separation properties, thus enabling the manipulation of protein and peptide sequences to design biocondensates with tailor-made properties.

## Supplementary Material



## Data Availability

The code underlying this study is openly available in our repository *UnderstandingLLPS* at https://github.com/BPSlab/UnderstandingLLPS.git.

## Notes

Amino acid enrichment across PhSePs, DPRs, and NODPRs (overall and by protein family); CIDER server parameter distributions for DPRs, NODPRs, and designed peptides; and motif presence and frequency as well as unique motifs identified for each protein family (PDF)
