# Supplementary material for: Understanding Biases in Liquid–Liquid Phase Separation: Investigating Amino Acid Enrichments in Phase-Separating Proteins toward Peptide Design
Source: Biomacromolecules. 2025 Sep 22;26(11):7247–64. doi: 10.1021/acs.biomac.4c00224 (PMC12818744; doi:10.1021/acs.biomac.4c00224)
Supplement: Supplementary file 1 [file bm4c00224_si_001.pdf]

Supplementary Information for

Understanding Biases in Liquid-Liquid Phase Separation: Investigating Amino acid Enrichments in Phase-Separating Proteins Towards Peptide Design

Joana Calvário [1], Diogo Antunes [1], Rita Cipriano [1], Daniela Kalafatovic [2,3], Goran Mauša [3,4], Ana S. Pina [1]*

1 Instituto de Tecnologia Química e Biológica António Xavier, Universidade Nova de Lisboa, Av. da República, 2780-157 Oeiras, Portugal

2. University of Rijeka, Faculty of Biotechnology and Drug Development, 51000 Rijeka, Croatia

3. University of Rijeka, Center for Artificial Intelligence and Cybersecurity, 51000 Rijeka, Croatia

4. University of Rijeka, Faculty of Engineering, 51000, Rijeka, Croatia

KEYWORDS: Membraneless Organelles, Liquid-Liquid Phase Separation (LLPS), Intrinsically Disordered Proteins (IDPs), Peptides, Motifs, Amino acid Enrichment, Multivalency

1. Supplementary Notes

1.1 Family-specific motif variations

In the general motif discovery phase, we identified 129 motifs with a CF $\geq$ 0.2. For the protein families, CF thresholds were adjusted to yield comparable motif counts: RNA binding (CF $\geq$ 0.35, 90 motifs), DNA binding (CF $\geq$ 0.30, 72 motifs), Chromatin binding (CF $\geq$ 0.40, 66 motifs), Regulation (CF $\geq$ 0.25, 74 motifs), Hydrolases (CF $\geq$ 0.40, 84 motifs), and Structure (CF $\geq$ 0.30, n=107).

2. Supplementary Figures

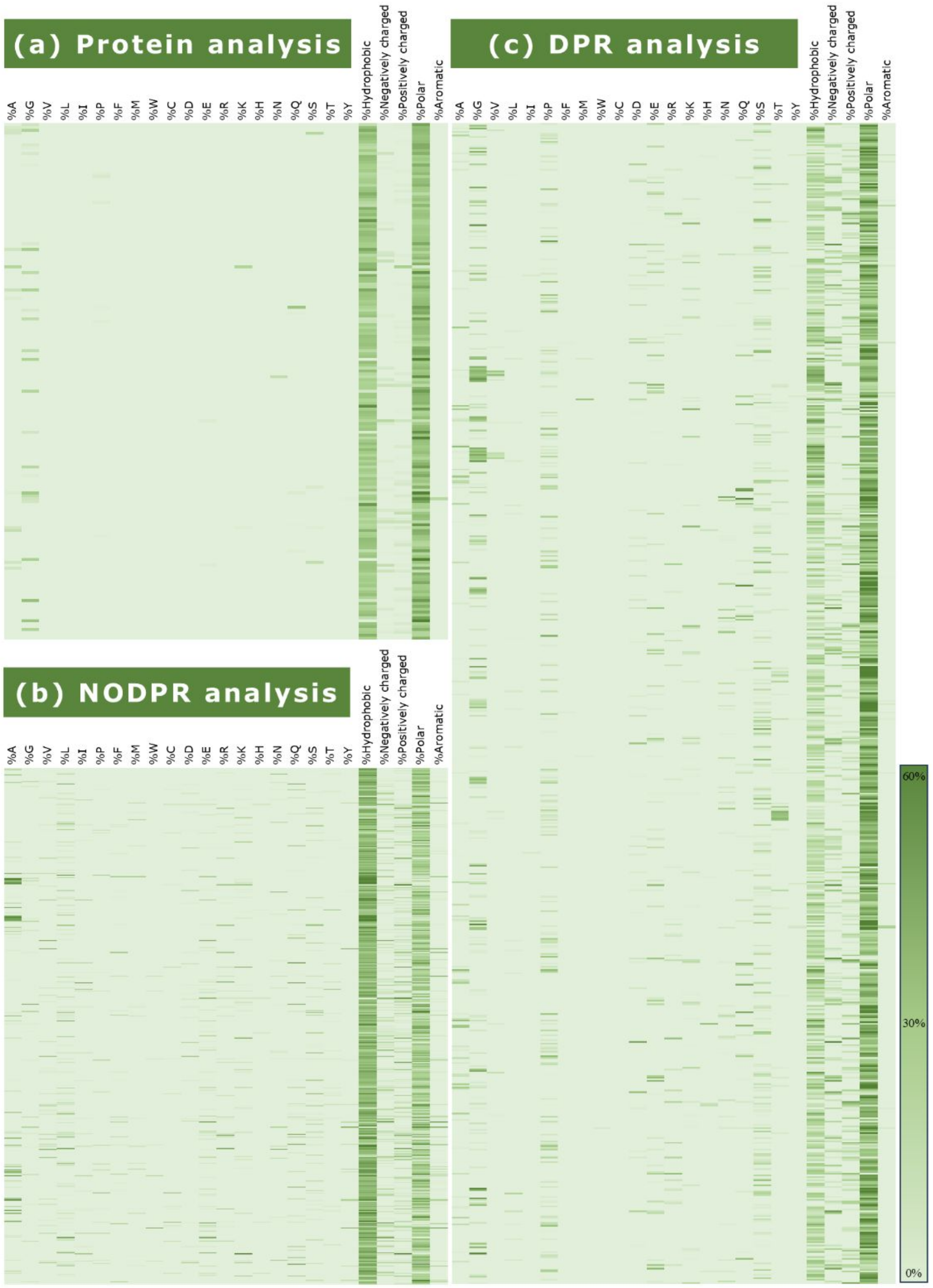

Figure S1 - Analysis of amino acids and residue character enrichment in (a) PhSePs; (b) NODPRs; (c) DPRs. The color gradient transitions from lighter hues (corresponding to 0%) to darker shades (indicating the maximum value observed at 60% presence).

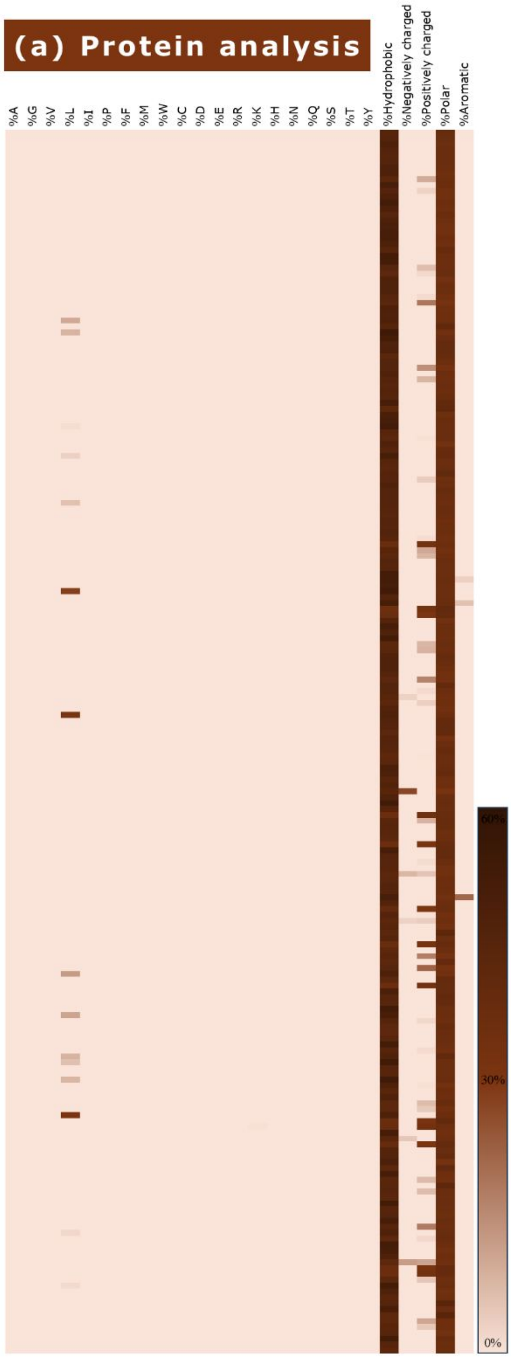

Figure S2 - Analysis of amino acids and residue character enrichment in (a) non-PhSePs. The color gradient transitions from lighter hues (corresponding to 0%) to darker shades (indicating the maximum value observed at 60% presence).

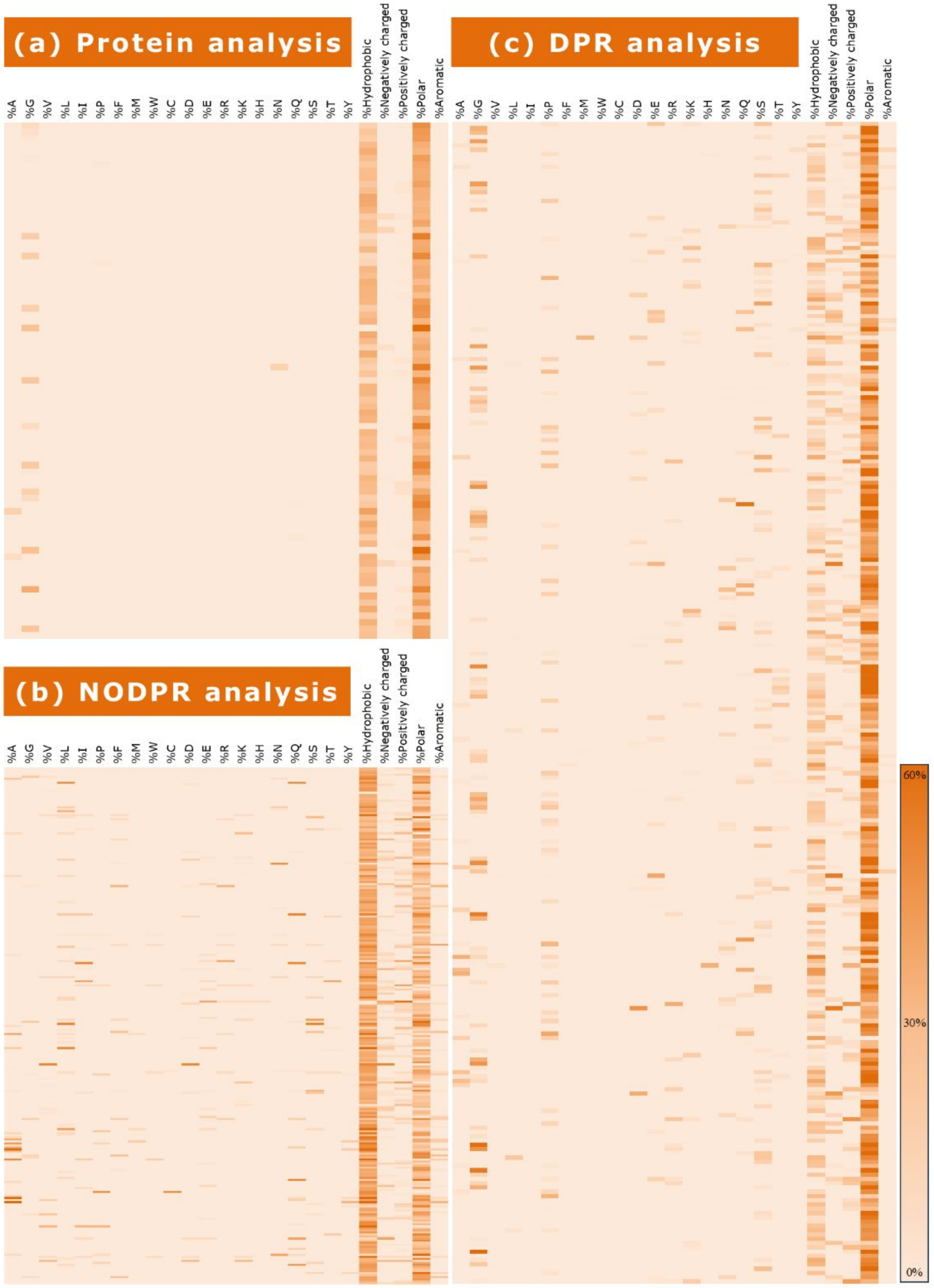

Figure S3 - Analysis of amino acids and residue character enrichment in the RNA binding family, (a) PhSePs; (b) NODPRs; (c) DPRs. The color gradient transitions from lighter hues (corresponding to 0%) to darker shades (indicating the maximum value observed at 60% presence).

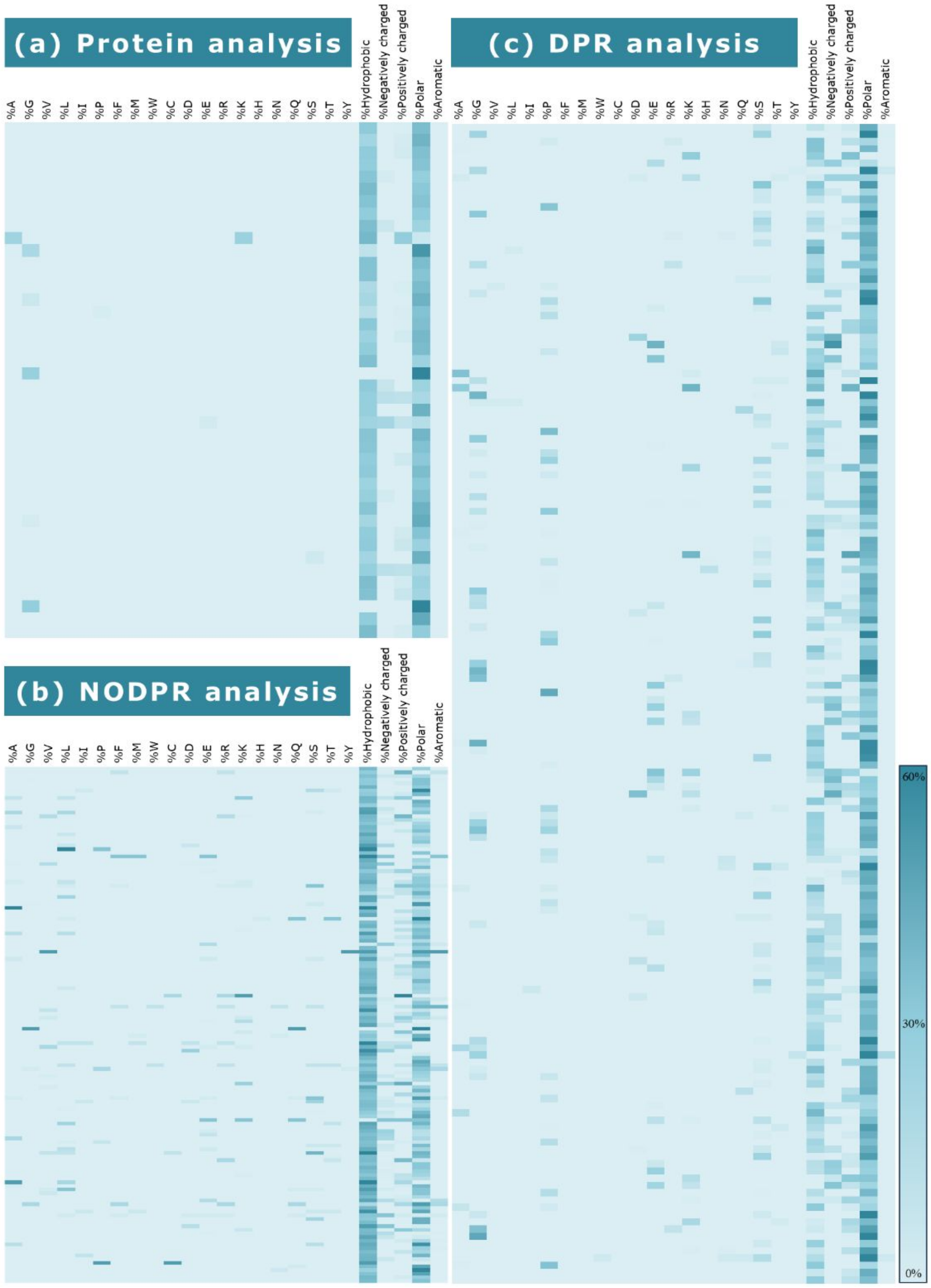

Figure S4 - Analysis of amino acids and residue character enrichment in the DNA binding family, (a) PhSePs; (b) NODPRs; (c) DPRs. The color gradient transitions from lighter hues (corresponding to 0%) to darker shades (indicating the maximum value observed at 60% presence).

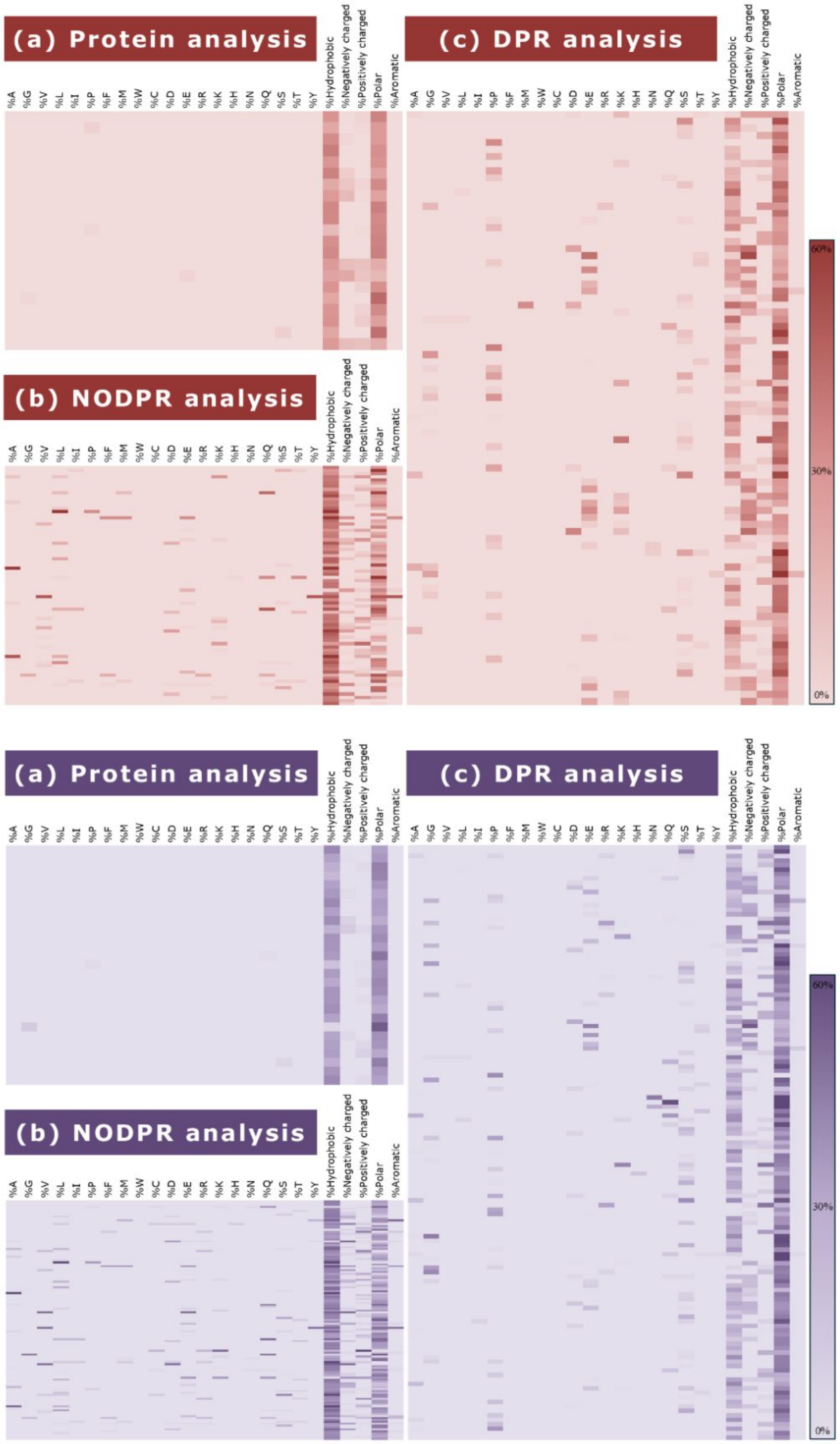

Figure S5 - Analysis of amino acids and residue character enrichment in the Chromatin binding (red) and Regulation (purple) families, (a) PhSePs; (b) NODPRs; (c) DPRs. The color gradient

transitions from lighter hues (corresponding to 0%) to darker shades (indicating the maximum value observed at 60% presence).

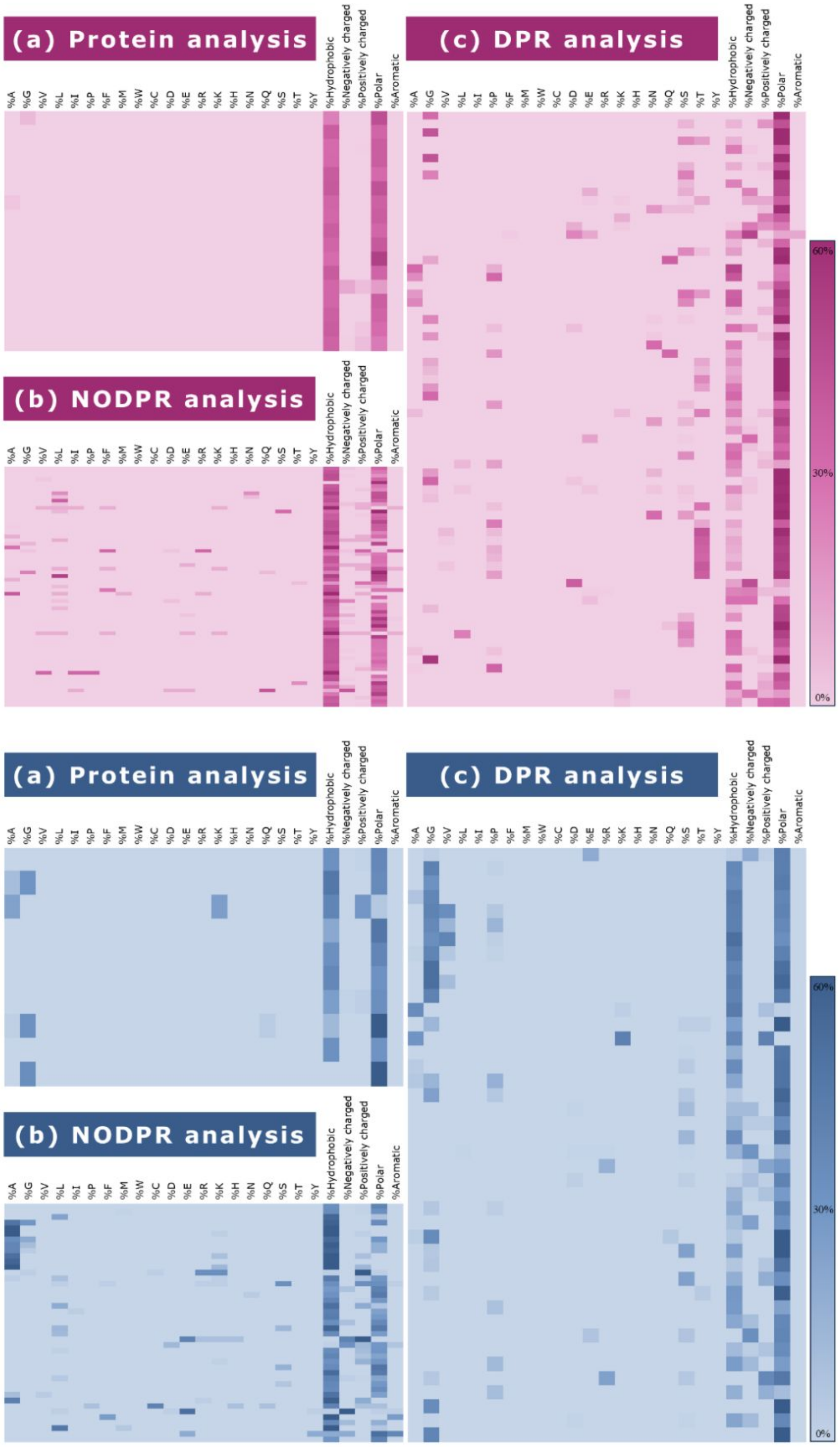

Figure S6 - Analysis of amino acids and residue character enrichment in the Hydrolase (pink) and Structure (dark blue) families, (a) PhSePs; (b) NODPRs; (c) DPRs. The color gradient transitions

from lighter hues (corresponding to 0%) to darker shades (indicating the maximum value observed at 60% presence).

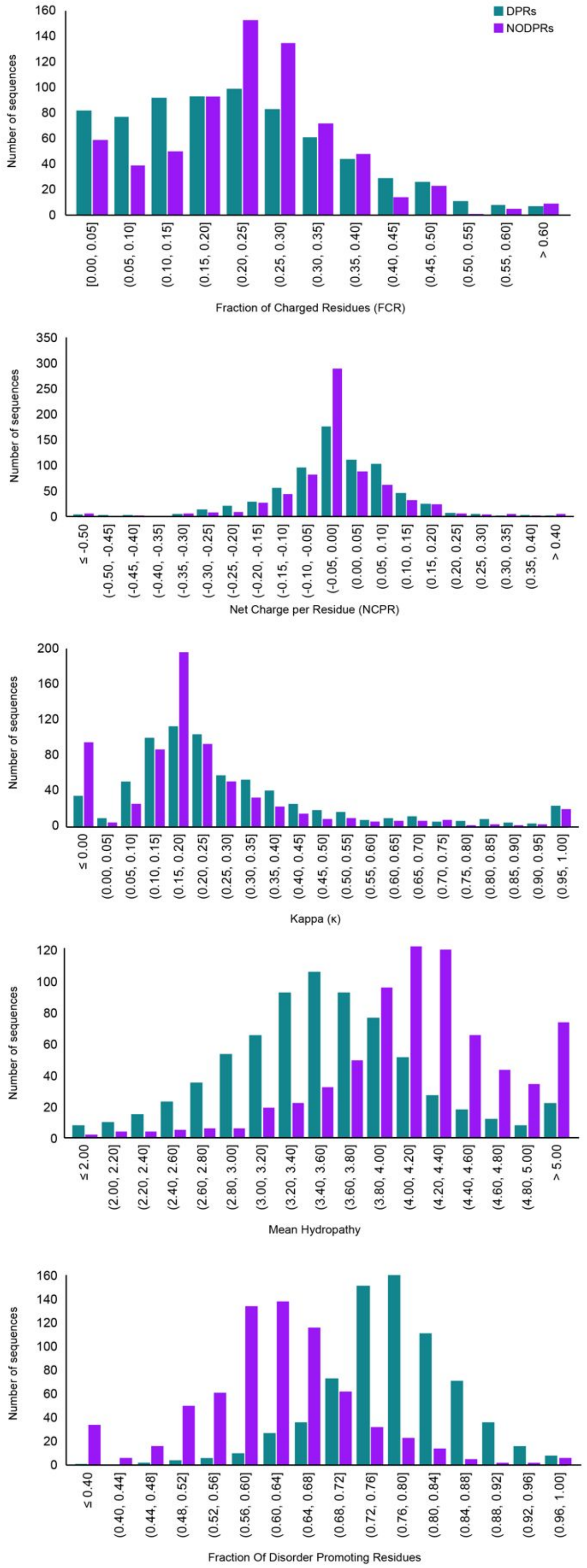

Figure S7 – CIDER Server parameter distribution for DPRs and NODPRs.

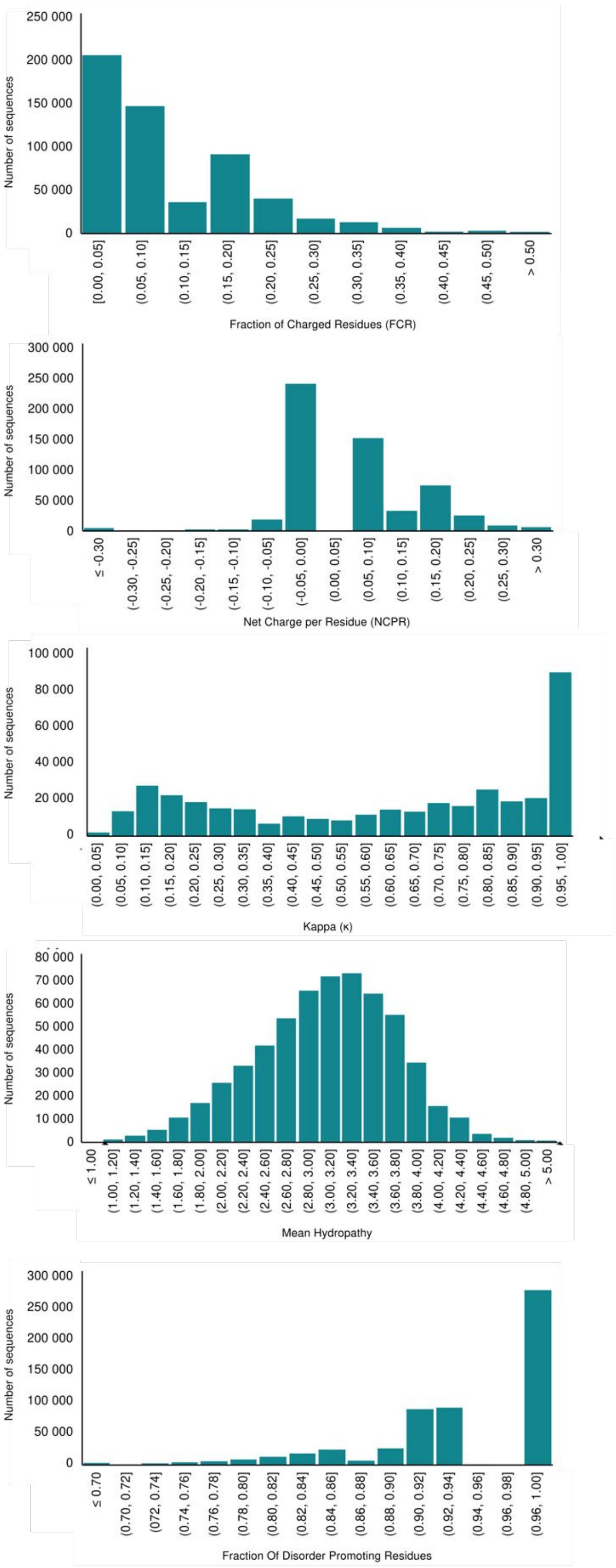

Figure S8 - CIDER Server parameter distribution for designed peptides.

3. Supplementary Tables

Table S1 – Presence and Frequency values of discovered motifs in DPRs and NODPRs.

Motif	NODPR		DPR	
	Presence	Frequency	Presence	Frequency
AAPA	1	1	13	23
DDED	1	1	11	12
DEDD	1	1	11	13
DRGG	0	0	13	39
DSSS	2	2	11	21
FGGG	1	1	13	20
GAPG	0	0	11	27
GDRG	0	0	14	39
GDRGG	0	0	11	32
GFGG	1	1	18	28
GGDR	2	2	13	44
GGDRG	0	0	12	37
GGDRGG	0	0	11	32
GGFGG	0	0	10	16
GGGG	1	1	40	98
GGGGF	0	0	10	13
GGGGG	1	1	25	51
GGGGGG	0	0	14	25
GGGGR	0	0	11	16
GGGGS	0	0	13	16
GGGN	2	2	14	29
GGGR	2	2	26	35
GGGRG	0	0	17	25
GGGRGG	0	0	14	16
GGGSG	0	0	15	20
GGGSGG	0	0	11	13
GGGY	0	0	15	32
GGGYG	0	0	13	22
GGGYGG	0	0	12	20
GGNG	0	0	10	19
GGPGG	0	0	10	13
GGPP	0	0	12	13
GGRG	1	1	29	70
GGRGG	0	0	22	46
GGRS	0	0	11	11
GGSGG	0	0	15	21
GGSS	2	2	12	15
GGYG	1	1	23	74
GGYGG	0	0	17	47
GNGG	1	1	8	19
GPGS	1	1	9	21
GPPP	0	0	16	22
GPYG	0	0	9	21
GRGG	0	0	34	86
GRGGG	0	0	15	25
GRGGY	0	0	10	14
GRGR	1	1	13	15
GRGS	0	0	10	13
GSGGG	0	0	10	14
GVPGV	0	0	8	19
GYGGG	0	0	9	16
GYGN	1	1	11	11
HHP	2	2	13	16
HQQQ	0	0	15	21
HQQQQ	0	0	12	16
PAPA	1	1	17	31
PGGG	1	4	16	25
PGGP	0	0	14	17
PGQQ	1	1	5	35
PGVG	1	2	18	43
PGVGV	1	1	8	23
PPPG	2	2	22	25
PPPP	4	4	38	70
PPPPP	0	0	19	35
PPPPPP	0	0	10	16

Table S1 - continued

Motif	NODPR		DPR	
	Presence	Frequency	Presence	Frequency
PPPPQ	0	0	11	14
PPPQ	0	0	22	35
PPQG	1	1	12	13
PPSS	0	0	14	15
PQQP	1	1	11	16
PQQQ	0	0	19	32
PQQQQ	0	0	14	20
PSGP	0	0	12	14
PSSS	2	2	12	17
PSYS	0	0	2	46
PSYSP	0	0	2	46
PSYSPT	0	0	2	42
PTSPSY	0	0	2	42
QGPG	1	1	4	46
QPN	3	3	15	18
QPPPP	1	1	12	18
QPQQ	1	1	12	18
QQGP	0	0	2	45
QQPP	2	2	17	28
QQPPP	1	1	11	18
QQPQ	0	0	13	18
QQQ	18	19	58	199
QQQH	1	1	10	18
QQQP	1	1	24	31
QQQQ	6	6	36	110
QQQQP	0	0	13	17
QQQQQ	4	4	28	64
QQQQQP	0	0	11	12
QQQQQQ	1	1	22	46
RGGD	0	0	12	16
RGGF	1	1	19	29
RGGFG	0	0	10	13
RGGG	1	1	26	50
RGGGG	0	0	14	24
RGGR	2	2	23	43
RGGRG	0	0	15	34
RGGRGG	0	0	11	24
RGRG	1	1	24	32
RGRGG	0	0	17	21
RRGG	1	1	13	16
SAPA	0	0	12	23
SGGGG	0	0	13	19
SGGGGG	0	0	11	15
SPSYS	0	0	2	46
SPSYSP	0	0	2	46
SPTSP	0	0	4	63
SPTSPS	0	0	3	41
SQQP	0	0	12	12
SRGG	1	1	19	20
SSAP	2	2	10	22
SSDS	1	1	13	16
SSSD	6	6	11	16
SSTG	2	2	11	14
SYSPT	0	0	2	42
SYSPTS	0	0	2	42
TSPSY	0	0	2	42
TSPSYS	0	0	2	42
VPGVG	0	0	9	22
VPPP	3	3	17	17
YGGG	0	0	14	30
YGPG	1	1	6	29
YSPT	0	0	2	61
YSPTS	0	0	2	60
YSPTSP	0	0	2	60

Table S2 – Unique motifs identified for each protein family.

RNA binding	DNA binding	Chromatin binding	Regulation	Hydrolase		Structure	
FRGGRG	GGSGG	ERRRRE	AQAQ	AASSS	NVGDTT	AAAK	GVGG
GDRGGF	GGSGGG	HHQ	AQAQA	ATPT	PATPT	AAAKK	GVGGL
GGFGG	GRGRG	KSKK	ASSPG	ATPTT	PATPTT	AAAKKK	GVGGLG
GGFGGG	GSGGG	MGQ	GGPGG	ATPTTP	PPHLR	AAAKKP	GVKP
GGFRGG	GSGGGG	NTWE	GGPGGP	DEDD	PTTP	AAGG	GVLPG
GGGGGF	GYRGRG	NTWEP	GPGGP	DRGGR	PTTPV	AAKK	GVLPGV
GGGGR	HHT	PKH	HPSSM	DRGGRG	PTTPVT	AAKKP	GVPG
GGR	PSYS	PPGA	PAPA	DRGR	PVTTP	AAKKPK	GVPGV
GGRGGD	PSYSP	PPP	PAPAP	DTT	TEPA	AAKP	GVPGVG
GGRGGY	PSYSPT	PPPG	PASSS	DTTE	TEPAT	AGG	KAAAKK
GGYG	PTSPSY	PPPH	PPASS	DTTEP	TEPATP	AGGY	KKDG
GGYGGD	RGRGGG	PPPLP	PPSS	DTTEPA	TGFG	AKKPKK	KKPKKA
GGYGGG	SPSYS	PPPPPP	PPTPP	EPATP	TGTA	APGAIP	KPA
GRG	SPSYSP	PPPPPQ	PPTT	EPATPT	TPTT	DENK	KPKA
GRGGDR	SPT	PPPPQ	PQQQ	GDRGGR	TPTTP	ETSK	KPKKA
GRGGF	SPTSP	PPPQQ	PQQQQ	GDTT	TPTTPV	GAGG	LGVGG
GRGGY	SPTSPS	PQM	PTPP	GDTTE	TPVTT	GAGGAG	LGVGGL
GYGGDR	SRGGG	PQQPPP	QAQA	GDTTEP	TPVTTP	GAKA	LPGVG
GYGGG	SRGGGG	QHH	QAQAQ	GGGGF	TTEP	GAPG	LPGVGV
HQQQQQ	SSFSSS	QPPPPP	QAQAQA	GGGGY	TTEPA	GAPGA	LSFG
MGG	SSSFSS	QQQPP	QQPQ	GGLFG	TTEPAT	GAPGAI	NGNG
MGM	SSSSST	REQ	QQPS	GGVNV	TTPV	GAVPG	NGNGG
NDN	SYSPT	SSDS	QRSA	GGVNVG	TTPVT	GFGPGG	PGAIPG
NYN	SYSPTS	SSSD	RGGPG	GLFG	TTPVTT	GGGN	PGAPG
PPM	TSPSY	SSSDS	RGGPGG	GVNVG	VGDTT	GGLG	PGAPGA
PPQ	TSPSYS	TPP	RGRGR	GVNVGD	VGDTTE	GGLGV	PGAVPG
RGGDRG	YRGRGG	TWE	RQQQQ	MYS	VNVGD	GGLGVG	PGG
RGGFG	YSPT	TWEP	SAAAA	NGGGG	VNVGDT	GGNG	PGGQ
RGGFGG	YSPTS	TWEPE	SHSR	NGGGGG	VTTP	GGNGN	PGGR
RGGGG	YSPTSP	WEPE	SPSSD	NVGD	YGGDRG	GGNGNG	PGGRP
RGGGGG			SQGEE	NVGDT		GGRP	PGGV
RGGYG			SQPQ			GGVGGL	PGGVAG
RGGYGG			SSHS			GGVPG	PGIG
YGG			SSTG			GLGV	PGLG
YGGG			TPPTT			GLGVG	PGQ
YND			TPTA			GLGVGG	PGQG
YQA						GLPG	PGVG
						GLPGV	PGVGV
						GLPGVG	PGVYPG
						GNG	PKKA
						GNGG	QQG
						GNGN	QSD
						GNGNG	SGGR
						GNGNGG	SGGRP
						GPGG	SGPGG
						GPGGQ	VGAGV
						GPY	VGGLG
						GPYG	VGGLGV
						GQGG	VKPK
						GRDG	VPGA
						GSGP	VPGVG
						GVGAG	VPGVGV
						GVGAGV	YGAP
